# The Antimicrobial and Antioxidant Properties of Raw, Aged, and Fermented Garlic: Influence of Processing Methods

**DOI:** 10.1002/fsn3.70743

**Published:** 2025-07-31

**Authors:** Luis M. Cocom, Hsiao‐Chi Wang, Kuo‐Chan Tseng, Yung‐Lin Chu

**Affiliations:** ^1^ Department of Tropical Agriculture and International Cooperation, International College National Pingtung University of Science and Technology Pingtung City Taiwan; ^2^ Department of Beauty Science, College of Health National Taichung University of Science and Technology Taichung Taiwan; ^3^ Department of Food Science, College of Agriculture National Pingtung University of Science and Technology Pingtung City Taiwan

**Keywords:** aged garlic, antimicrobial, antioxidation, organosulfur compounds

## Abstract

Garlic ( *Allium sativum* L.) is widely recognized for its bioactive properties, primarily attributed to its sulfur‐containing compounds (SCs), which provide both prophylactic and therapeutic benefits. This study evaluates the antimicrobial and antioxidant activities of raw, aged, and fermented garlic, utilizing ultrasound‐assisted extraction (UAE). Processed garlic samples, including fermented garlic in fruit vinegar (FGV), honey (FGH), ethanol (FGE), aged garlic (AGE60), and fresh raw garlic (RAW), were analyzed to determine the effects of different processing methods on their functional properties. Antimicrobial activity was assessed using the agar well diffusion method to measure inhibition zones (IZ) and microdilution techniques to determine the minimum inhibitory concentration (MIC) against *Escherichia coli* , *Aspergillus niger*, and *Pichia guilliermondii*. The results indicate that FGV, FGH, FGE, and AGE60 exhibited notable antimicrobial activity, while RAW garlic demonstrated the strongest antimicrobial effects, primarily due to its high allicin content and other sulfur‐containing organosulfur compounds (OSCs), which are recognized as potent antimicrobial agents. Antioxidant capacity was evaluated through free radical scavenging activity (RSA) using the 1,1‐diphenyl‐2‐picrylhydrazyl (DPPH) assay, ferric reducing antioxidant power (FRAP), and total phenolic content (TPC). AGE60, FGH, and FGV exhibited the highest antioxidant activity, with fermentation and aging processes contributing to the production of flavonoids and phenolic compounds, thereby enhancing antioxidative capacity. However, these processing methods did not significantly improve antimicrobial properties. These findings highlight the impact of processing methods on garlic's functional properties, suggesting that different processing techniques may be tailored to optimize specific health benefits.

## Graphical abstract

The samples of FGV, FGH, FGE, and AGE60 exhibited notable antimicrobial activity, but fresh raw garlic (RAW) displayed the strongest antimicrobial properties. The fermentation and aging processes (AGE60, FGH, and FGV) contributed to the production of flavonoids and phenolic compounds, enhancing the best antioxidative properties among garlic samples in this study.

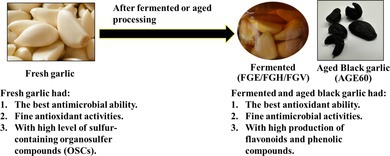

## 1 Introduction

Garlic ( *Allium sativum* L.) is widely used in culinary applications and traditional medicine, contributing to the growing global demand for medicinal plants. As of 2021, global garlic production exceeded 28 million metric tons (FAO 2021). Its distinctive nutritional profile is primarily attributed to its diverse phytochemical composition, which plays a crucial role in diet‐based therapeutic interventions for mitigating various lifestyle‐related disorders (Daliri et al. 2019; Itrat et al. 2024). Given its bioactive potential, advancements in garlic research may be extended to other food sources, including fruits and vegetables, to enhance consumer health and nutrition.

Raw garlic is a rich source of essential trace metals, including zinc (556.1 mg), manganese (446.9 mg), copper (143.3 mg), selenium (5.5 mg), and iodine (2.5 mg) per 100 g of fresh weight. Its protein content is relatively low, ranging from 2.6% to 3.0%, depending on the garlic variety and cultivation practices, with an average amino acid composition of approximately 2.13%. Additionally, fresh garlic's dietary fiber and total tocopherol content are approximately 2310 and 103.1 mg per 100 g of fresh weight, respectively. Beyond these nutrients, garlic is a significant source of various bioactive compounds, including saponins, polysaccharides, and polyphenolic compounds (Kasprzak‐Drozd et al. 2022; Skoczylas et al. 2023). Previous studies have characterized the chemical structures of several key bioactive sulfur‐containing compounds found in raw and processed garlic, such as diallyl sulfide (DAS), diallyl disulfide (DADS), diallyl trisulfide (DATS), diallyl thiosulfonate (allicin), S‐allyl‐cysteine sulfoxide (alliin), E/Z‐ajoene, and S‐allyl‐cysteine (SAC) (Geddo et al. 2023; Li et al. 2022; Reiter et al. 2020; Zhu et al. 2022). These bioactive components exhibit various medical benefits. Moreover, some plant species, such as *Mentha pulegium* , contain functional components similar to those found in garlic (Tanavar et al. 2021).

Alternative biochemical transformations and microbiota changes occur during garlic storage, differing from previously discussed processing methods (Skoczylas et al. 2023; Tonog et al. 2024). Specifically, prolonged immersion of intact or sliced raw garlic in solvents such as alcohol or vinegar induces the formation of distinct sulfur‐containing compounds, which differ significantly from those found in distilled garlic oil. Aged garlic processing results in a substantial loss of allicin, with *γ*‐glutamyl transpeptidase playing a critical role in the metabolic conversion of precursor sulfur‐containing compounds, such as *γ*‐glutamyl cysteine, into the hydrophilic compound *S*‐allyl cysteine (SAC). SAC subsequently undergoes further metabolic transformation into bioactive derivatives, including *S*‐allyl mercaptocysteine (SAMS) and *S*‐methyl cysteine (Hill et al. 2025; Zhang et al. 2014).

Aging is a key process examined in this study, with aged black garlic produced through the fermentation of fresh garlic cloves under controlled temperature, humidity, and duration conditions. This process gradually darkens the cloves, enhances their sweetness, and alters their texture to a chewier, jelly‐like consistency. Previous studies indicate that thermal processing and high hydrostatic pressure induce significant biochemical transformations in garlic‐derived compounds (Zhao et al. 2021). Specifically, Amadori compounds, derived from glucose‐amino acid interactions, and Heyns compounds, derived from fructose‐amino acid interactions, serve as intermediates in the Maillard reaction.

Fermentation plays a crucial role in developing numerous food products and beverages initiated through methods such as starter cultures, back‐slopping, or spontaneous fermentation. This process offers several advantages, including extended shelf life, improved microbial safety, and enhanced flavor, palatability, and overall organoleptic properties. In this study, spontaneous fermentation was employed, wherein environmental conditions facilitated the growth of indigenous microflora, which interacted with the raw material to drive fermentation. Moreover, previous research has reported that black garlic exhibits multiple beneficial functions, including anti‐inflammatory and anti‐obesity effects (Wu et al. 2020).

The Food and Agriculture Organization (FAO) defines vinegar as any liquid derived from starch and/or sugar‐containing products through a double‐stage fermentation process, initially alcoholic, followed by acetous, rendering it suitable for human consumption. Vinegar is among the few widely used acidic condiments and is a by‐product of ethyl alcohol fermentation into acetic acid by *Acetobacter* or acetic acid bacteria (AAB). In Europe, vinegar has historically served as a preservative in food, particularly for fruits, garlic, and vegetables in traditional markets.

Honey, on the other hand, is one of the most extensively consumed natural products in the food industry, primarily utilized as a sweetener. As a genuine biological product produced by honeybees from nectar, scientific studies have demonstrated its diverse health benefits, including gastro‐ and hepatoprotective, reproductive, hypoglycemic, antioxidant, and antimicrobial activities, making it a valuable supplement and alternative medicine. Honey contains high concentrations of primary sugars, such as oligosaccharides, monosaccharides, disaccharides, and polysaccharides (Cai et al. 2021; Tian et al. 2023). In addition, honey has been employed in food preservation processes. Therefore, vinegar and honey represent key processing methods examined in this study.

Garlic offers a wide range of health benefits, particularly its notable antibacterial and antifungal properties, largely attributed to its diverse sulfur‐containing compounds (Osaili et al. 2022; Serrano‐Jara et al. 2023). Among these, allicin, a thiosulfinate compound, has been identified as a key contributor to garlic's antimicrobial activity (Hassanzadeh et al. 2023; S. Li et al. 2024). Furthermore, diallyl trisulfide (DATS) and ajoene, organosulfur derivatives of allicin, have demonstrated antimicrobial efficacy in *previous* studies (Tang et al. 2021). However, despite these promising findings, robust evidence from randomized controlled trials evaluating garlic's antimicrobial efficacy in humans remains limited (Gail et al. 2007; Martin and Ernst 2003; You et al. 2006). A key focus of antimicrobial research is the inhibition rate of pathogenic bacteria. Previous studies have used similar bacterial strains to assess garlic's antimicrobial potential (Alizadeh Behbahani and Imani Fooladi 2018).

Antioxidants are molecules capable of inhibiting the oxidation of other molecules. In food systems, they are present in minimal concentrations yet effectively reduce or prevent the detrimental effects of reactive oxygen species (ROS) on normal physiological functions by interacting with oxidized substrates (Sinenko et al. 2021). Antioxidants can be broadly classified into two categories: primary antioxidants, which function directly to neutralize free radicals, and secondary antioxidants, which are distinguished by their enzymatic or non‐enzymatic modes of production (Stone et al. 2025). It has been explained that redox reactions can generate free radicals, initiating a chain reaction within cells that may lead to oxidative damage or apoptosis (Zhang et al. 2020). Consequently, antioxidants serve as a crucial defense mechanism, mitigating the harmful effects of oxidative stress. Garlic contains a diverse composition of antioxidants, encompassing polyphenolic compounds such as phenolic acids and flavonoids, as well as other bioactive classes, such as thiols, carotenoids, organosulfur compounds, minerals, and vitamins. In addition, previous research has indicated that processing methods modify the bioactive components of garlic, potentially influencing its antioxidant properties (Tedeschi et al. 2023).

This study aims to evaluate the effects of processed garlic extracts on antimicrobial, antioxidant, and anticancer activities. The samples include fresh, fermented (using 95% ethanol, fruit vinegar, and longan honey), and aged (at 60°C with 95% relative humidity) garlic. Additionally, this study aims to identify the optimal processing method that maximizes efficacy based on in vitro assessments of antimicrobial and antioxidant properties.

## 2 Materials and Methods

### 2.1 Chemicals and Reagents

Fresh garlic cloves, longan honey ( *Dimocarpus longan* ), 95% ethanol, and vinegar were procured from the National Pingtung University of Science and Technology. DMEM medium, Trypsin, 1× PBS, Griess reagent, NaNO_2_^−^, MTT 3‐(4, 5‐dimethylthiazole‐2‐yl)‐2, 5‐diphenyl tetrazolium bromide, DMSO, BSA, RIPA buffer, and sodium dodecyl sulfate‐polyacrylamide gel electrophoresis (SDS‐PAGE) reagents were purchased from Sigma.

### 2.2 Preparation of Processed Garlic and Extracts

Fresh garlic was obtained from the National Pingtung University of Science and Technology market and divided into six groups of 100 g each. To produce aged black garlic, fresh garlic was placed in an oven at 60°C for 6 months, with temperature and relative humidity carefully monitored and maintained at 60°C and 95%, respectively. The remaining garlic groups were peeled and placed in empty glass jars containing different fermentation mediums: 95% ethanol, longan ( *Dimocarpus longan* ) honey, and fruit vinegar (pH 3.5), undergoing fermentation for 6 months. Garlic extracts were prepared following the method of Mathialagan et al. (2017) with slight modifications. Specifically, 10 g of garlic was weighed and mashed using a mortar and pestle with 100 mL of ddH_2_O for the antimicrobial and antioxidant assessments. The resulting garlic matrix was incubated in a water bath at 30°C for 30 min for ultrasound‐assisted extraction. After sonication, the matrix was centrifuged at 5000 rpm at 4°C for 10 mins. The extract was then filtered through No. 1 Whatman filter paper and stored at 4°C before use. For the anticancer experiment, garlic samples were dried in an oven at 60°C for 24 h, then ground using a mortar and pestle, filtered, and stored at −20°C until further use. A serum‐free medium was utilized for extract preparation during treatment applications.

### 2.3 Microbial Strains and Inoculum Preparation

Each microbial organism was cultured and grown on its respective broth and agar medium. *Escherichia coli* ( *E. coli* ) was cultured in nutrient broth (NB), *Aspergillus niger* ( *A. niger* ) in Potato Dextrose broth (PDB), and *Pichia guilliermondii* (*P*. *guilliermondii*) in Yeast Malt broth (YMB).

### 2.4 Bacteria ( *Escherichia coli* NPUST 23)

*E. coli* stock was obtained from the Department of Food Science and thawed at room temperature. Using a 200 μL pipette, 0.1 mL of stock culture was transferred into a test tube containing 5 mL of NB. The broth was then incubated at 37°C for 24 h. After incubation, a 1 mL aliquot was pipetted into another test tube containing 9 mL of NB and mixed for 1 min. The inoculum is stored at 4°C and prepared fresh before each experiment.

### 2.5 Fungi (*Aspergillus niger*)

The mold stock was thawed at room temperature for about 20–30 min. To initiate inoculation, 0.1 mL of stock culture was transferred into a test tube containing 5 mL of PDB. The tube was incubated at 30°C for 2 to 5 days while being continuously stirred at 100 RPM. During incubation, an oblique slant of Potato Dextrose Agar (PDA) was prepared. After incubation, fungal cells were streaked from PDB to PDA using an inoculating loop, followed by incubation under identical conditions.

### 2.6 Yeast (*Pichia guilliermondii* Y23)

Yeast stock was thawed at room temperature before being streaked onto Yeast Malt Agar (YMA) in a 90 mm Petri dish. The plates were incubated at 30°C for 2–5 days. To prepare the inoculum, the yeast colony was washed with 5 mL of Yeast Malt Broth (YMB) or collected using an inoculating loop, resuspended in 5 mL YMB, stirred at 100 RPM, and incubated under the same conditions.

### 2.7 Antimicrobial Susceptibility Testing (AST)

#### 2.7.1 Inoculum Calibration (0.5 McFarland)

Following the Clinical and Laboratory Standards Institute (CSLI) guideline M7‐A9, five one‐day‐old bacteria ( *E. coli* ) colonies were aseptically transferred from nutrient agar into 5 mL of 0.85% saline solution. The suspension was vortexed for 15 s and adjusted to 0.5 McFarland standard. Using a 96‐well plate, 200 μL of the inoculum was dispensed into wells, and turbidity was measured at 625 nm using an ELISA reader. The optimal density (OD) range was 0.08 to 0.12, corresponding to a bacterial suspension of 1–2 × 10^8^ CFU/mL (CLSI 2012).

For yeast calibration, the CSLI guideline M27‐A2 was followed. Five one‐day‐old *P. guilliermondii* colonies (1 mm in diameter) were aseptically transferred into 5 mL of 0.85% saline solution and vortexed. Then, 200 μL of suspension was loaded into a 96‐well plate and measured at 530 nm, with OD ranging from 0.09 to 0.30. The suspension was adjusted using 0.85% saline solution to obtain a 0.5 McFarland standard. The stock microbial suspension was maintained at 5 × 10^4^ CFU/mL (CLSI 2002).

For filamentous fungi like *A. niger* , the broth microdilution method was conducted following CLSI guideline M38‐A2 (CLSI 2008). The fungal surface was treated with 0.4% Tween water and gently probed with a cotton swab. The suspension was filtered using a 0.22 μm nylon filter, and turbidity was measured at 530 nm with an OD range of 0.09 to 0.30. The final inoculum suspension for broth microdilution ranged from 0.4 × 10^4^ to 5 × 10^4^ and 0.4 × 10^6^ to 5.5 × 10^6^ CFU/mL. The CLSI guideline M51‐A was used to determine inhibition zones on a Petri dishes (CLSI 2010).

#### 2.7.2 Preparation of Controls

The positive control varied based on the tested microorganism. In the case of *E. coli* , amoxicillin was used at a minimum concentration of 10 μg/mL. For yeast (*P. guilliermondii*) and fungus ( *A. niger* ), Clotrimazole was used at 50 mg/mL, diluted in Dimethyl Sulfoxide (DMSO) and further adjusted to 16 μg/mL. A 0.85% saline solution was used as the negative control.

#### 2.7.3 Agar Well Diffusion Method

Antimicrobial susceptibility was assessed using the agar well diffusion method (Hossain et al. 2022) to evaluate the antimicrobial activity of processed garlic extracts. The target microorganism was inoculated in a liquid medium (NB, PDB, or YMB), followed by serial dilutions to obtain the desired CFU/mL for inoculation. A bacterial concentration of 7 × 10^−6^ CFU/mL was used to inoculate the sterile agar surface achieved by spreading 100 μL of microbial inoculum across the agar surface. Wells (6–8 mm in diameter) were aseptically punched using a flamed metal cork borer, and 100 μL of garlic extract and control solutions were introduced into each well. Plates were incubated under appropriate conditions: 12 h for *E. coli* and 24 h for *A. niger* and *P. guilliermondii*. Following incubation, inhibition zones were measured in millimeters.

#### 2.7.4 Minimum Inhibitory Concentration (MIC)

MIC represents the lowest concentration of an antimicrobial agent that prevents visible microbial growth, expressed in mg/L (μg/mL) (Jalil Sarghaleh et al. 2023). The MIC of processed garlic extracts was determined using a microdilution assay in a 96‐well round‐bottom plate, following CSLI guidelines with modifications. According to the CSLI guidelines M7‐A9 (CLSI 2012), M27‐A2 (CLSI 2002), and M38‐A2 (CLSI 2008), 50 μL of the growing medium specific to each microorganism was loaded into each well. Subsequently, 100 μL of each garlic extract was loaded into well 1 and serially diluted by transferring 50 μL across wells 1–10. The final 50 μL from well 10 was discarded. A 10 μL inoculum suspension, prepared at an adequate density, was then added sequentially from well 11 to 1. Wells 12 (A‐H) served as negative controls without microorganisms.

#### 2.7.5 Antioxidant Activity

The following reagents were utilized for the assessment of antioxidant activity: 2,2‐diphenyl‐1‐picrylhydrazyl (DPPH), ferrous sulfate heptahydrate, sodium acetate, acetic acid, 2,4,6‐tripyridyl‐s‐triasine (TPTZ), hydrochloric acid (HCl), ferric trichloride (FeCl_3_), sodium carbonate (Na^2^CO_3_), gallic acid, aluminum chloride (AlCl_3_), sodium nitrite (NaNO_2_, 5%), sodium hydroxide (NaOH, 1 M), ethanol (EtOH, 75%), methanol (MeOH, 100%), sodium phosphate buffer (0.2 M, pH 6.6), and potassium ferricyanide (1%).

#### 2.7.6 Free Radical Scavenging Activity (RSA) – DPPH

The free radical scavenging activity (RSA) of garlic extracts was assessed using the Blois (1958) method with minor modifications, as described by (Hu et al. 2023). A volume of 50 μL of 0.1 mmol 2, 2‐diphenyl‐1‐picrylhydrazyl (DPPH) in 75% ethanol was added to 100 μL of each tenfold diluted sample. The mixture was thoroughly stirred and incubated at room temperature (RT) in the dark for 30 min. Absorbance measurements of the garlic extract samples were obtained at 517 nm (OD_517_) using an ELISA kit, with all experiments conducted in triplicate. The DPPH RSA was calculated as a percentage using Equation (1):

(1) $$\text{DPPH}\;\mathrm{RSA}\;\left(\%\right)=1-\left(\frac{{\mathrm{OD}}_{\text{sample}}}{{\mathrm{OD}}_{\text{control}}}\right)\times 100$$

where, OD_sample_ represents the optical density of the sample, and OD_control_ denotes the optical density of the control.

#### 2.7.7 Ferric Reducing Antioxidant Power (FRAP) Assay

The antioxidation activity of the garlic extracts, assessed through ferric‐reducing power, was evaluated using the FRAP assay as described by Benzie and Strain (1996) and Hu et al. (2023). Iron‐reducing capacity was determined by adding 10 μL of ferrous sulfate (FeSO_4_) to establish a standard curve at varying concentrations (0, 250, 500, 750, 1000, 1250, 1500, 1750, and 2000 μM) or by using the garlic extract sample. Each reaction was performed in triplicate in a 96‐well round‐bottom plate. Subsequently, 150 μL of FRAP reagent was added.

The FRAP reagent was prepared in a 10:1:1 volumetric ratio, consisting of 300 mmol of acetate buffer (pH 3.6), 10 mmol TPTZ in 40 mmol HCl, and 20 mmol ferric chloride hexahydrate (FeCl_3_6H_2_O) following the method by (Li et al. 2018). DD H_2_O served as the blank control. The reaction mixture was covered with aluminum foil and incubated in the dark at RT for 30 min. Absorbance was measured at 593 nm (OD_593_).

FRAP values were calculated using Equation (2), expressed as μmol of ferrous sulfate equivalent per liter (FeSO_4_ μmol/L).
(2)

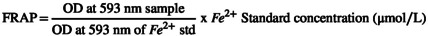




#### 2.7.8 Total Phenolic Content (TPC) Assay

The total polyphenolic content (TPC) of garlic extracts was assessed using the Folin–Ciocalteu reagent with minor modifications. Gallic acid was used to establish the standard curve, prepared as a stock solution at 500 μg/mL and subsequently diluted to final concentrations of 0, 50, 100, 150, 200, 250, and 300 mg/L. A volume of 20 μL of either the garlic extract or gallic acid standard was loaded into a 96‐well plate. Next, 100 μL of Folin–Ciocalteu reagent was added, and the mixture was allowed to react for 5 min. Subsequently, 80 μL of a 7.5% sodium carbonate solution was added, and the reaction mixture was thoroughly mixed. DD H_2_O was used as the blank control. The plate was then covered with aluminum foil and incubated in the dark at RT for 30 min. Absorbance measurements were obtained at 765 nm (OD_765_) in triplicate using a microplate reader. The phenolic content of the extracts was expressed as gallic acid equivalent (mg GAE/g) following the methodology described by Hu et al. (2023) and Hsu et al. (2024).

### 2.8 Statistical Analysis

All values are presented as mean ± SD based on triplicate measurements. Statistical significance was assessed using one‐way analysis of variance (ANOVA), followed by Tukey's post hoc multiple comparison test at a significance level of *p* < 0.05. All statistical analyses were performed using IBM SPSS Statistics version 25.0. Additionally, correlation analysis was conducted using Pearson's correlation test to evaluate relationships between sample variables following the methodology described by (Hsieh et al. 2023; Sunthorn et al. 2025).

## 3 Results and Discussion

### 3.1 Antimicrobial Activity Assessment

This study assessed the antimicrobial ability of five garlic extracts using the agar well diffusion method, a widely recognized technique for assessing plant‐based antimicrobial activity. This method is cost‐effective, reproducible, and easy to interpret, making it well‐suited for preliminary screening. However, its primary limitation is the inability to yield the minimum inhibitory concentration (MIC), which is essential for quantifying antimicrobial efficacy. Hence, the MIC of all garlic extracts in this study was determined using the microdilution technique.

**FIGURE 1:**
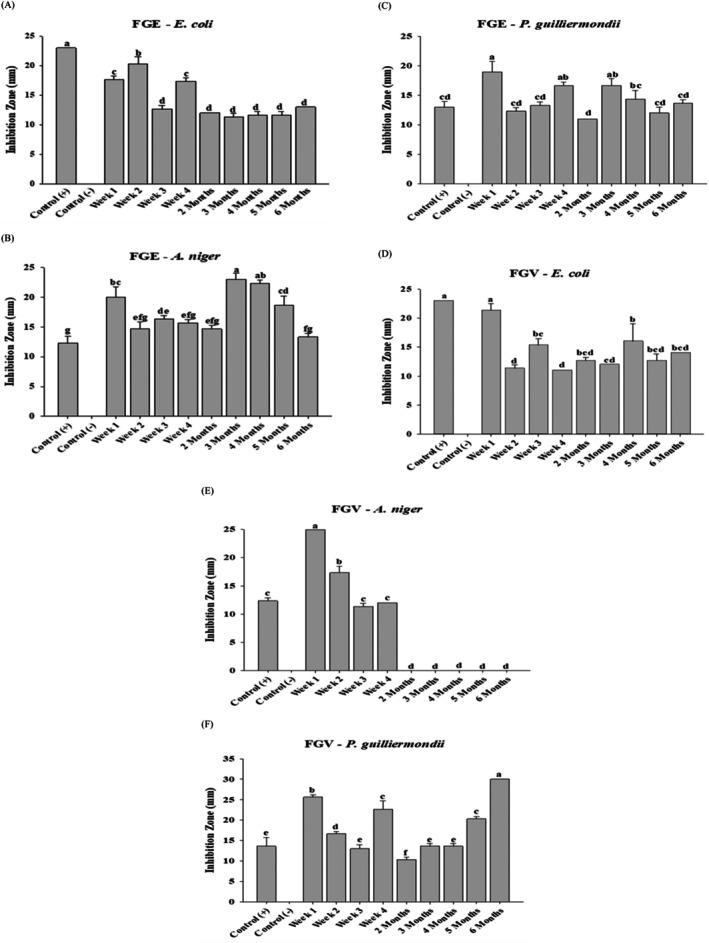
The comparative antimicrobial analysis of different kinds of fermented garlic against selected microorganisms. Antimicrobial activity of garlic fermented in 95% ethanol (FGE) on *E. coli* (A), *A. niger* (B), and *P. guilliermondii* (C), and garlic fermented in fruit vinegar (FGV) on *E. coli* (D), *A. niger* (E), and *P. guilliermondii* (F). The results are represented as means ± SD of triplicate independent readings. Values carried with different letters show significantly different (*p* < 0.05).

Inhibition zone (IZ) for FGE was consistently lower than that of the control group. The highest IZ for *E. coli* (Figure 1A) was observed at week 2, measuring 20.3 ± 1.2 mm, while the lowest was observed after week 4 at 11.3 ± 0.6 mm. After week 4, the IZ remained relatively stable until the final phase of the experiment at 6 months. In the case of *A. niger* (Figure 1B), the highest IZ of 23.0 ± 1.0 mm was observed at 3 months, followed by a decline at 6 months to 13.3 ± 0.6 mm, nearing the control group values. This suggests that FGE exhibits a strong antimicrobial effect on *A. niger* during the later stages of fermentation. Conversely, FGE displayed less effectiveness against *P. guilliermondii* (Figure 1C), with its highest IZ at 19.0 ± 1.7 mm at the end of week 1 and the lowest at 11.0 ± 0.0 mm by the end of the second month, which was lower than that of the control. Table S1 demonstrates the inhibition zones of garlic fermented in 95% ethanol extract at different processing durations. In addition, the MIC was determined throughout the fermentation process. The MIC varied significantly over different time points, rendering precise determination challenging. Specifically, for *E. coli* , the MIC at 3 and 6 months was zero.

The highest IZ for *E. coli* (Figure 1D) peaked at 23.0 ± 0.0 mm at the end of week 1, whereas the lowest was observed after weeks 2 and 4, measuring 11.0 ± 0.0 mm (*p* ≤ 0.05). The IZ fluctuated notably across fermentation periods, consistently remaining below control values. For *A. niger* (Figure 1E), the highest IZ was observed at the end of week 1 at 25.0 ± 0.0 mm, followed by a sharp decrease to 12.0 ± 0.0 mm by week 4. After 4 weeks, no IZ was detected, suggesting that the efficacy of FGV diminished beyond this period.

FGV exhibited a more favorable antimicrobial effect on *P. guilliermondii* (Figure 1F), with the highest IZ recorded at month 6 (30.0 ± 0.0 mm) and the lowest at the end of the second month (10.3 ± 0.6 mm). A distinct pattern was observed, where IZ initially declined from week 1 but gradually increased from month 3, peaking at month 6. Table S2 summarizes the antimicrobial activity of garlic fermented in fruit vinegar, including MIC values. For *E. coli*, the MIC recorded at the end of week 1 was 3.1 mg/L. The MIC for *A. niger* was 8.8 mg/L at week 1, while *P. guilliermondii* exhibited a MIC of 15.6 mg/L at 6 months.

In summary, garlic fermented in fruit vinegar (FGV) for 6 months showed antimicrobial activity similar to FGE against *E. coli, A. niger*, and *P. guilliermondii*. Across all control groups, the average IZ values were 23.0 ± 0.0, 12.3 ± 0.6, and 13.7 ± 2.1 mm, respectively. The negative control, consisting of 0.85% saline solution, showed no inhibitory effects against any of the tested microorganisms.

**FIGURE 2:**
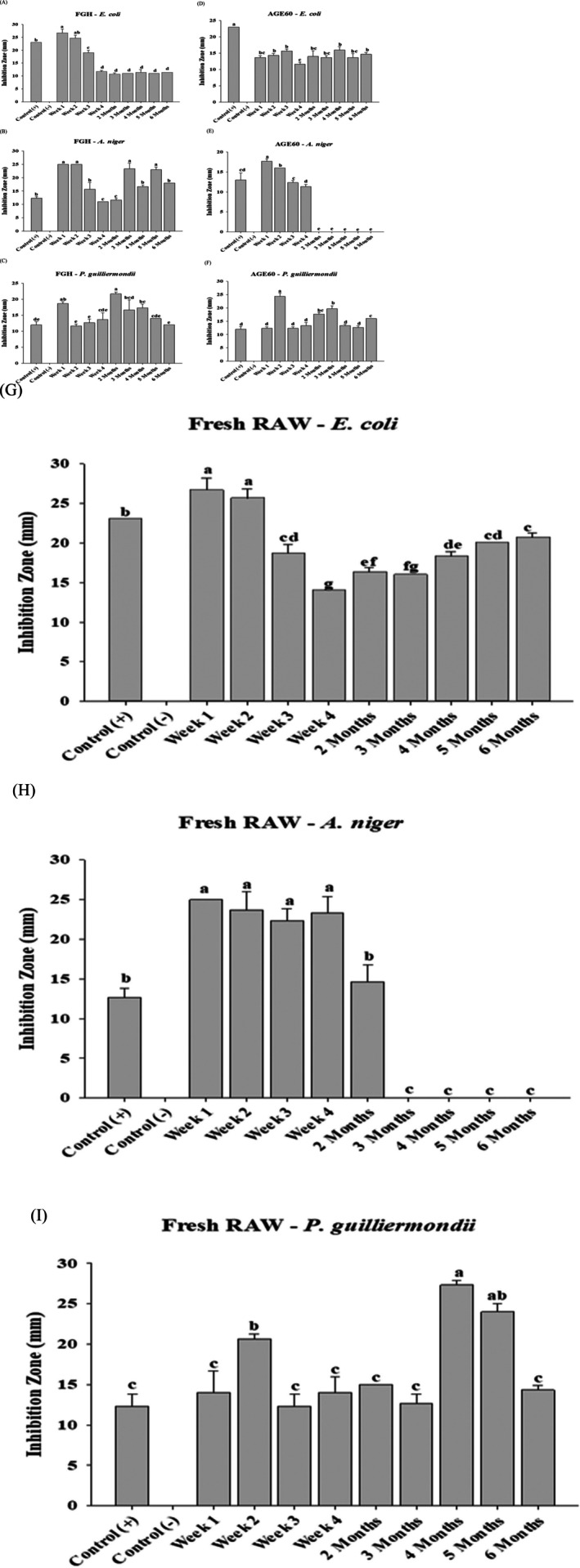
The comparative antimicrobial analysis of different kinds of fermented garlic against selected microorganisms. Antimicrobial activity of garlic fermented in honey (FGH) on *E. coli* (A), *A. niger* (B), and *P. guilliermondii* (C), aged black garlic (AGE60) on *E. coli* (D), *A. niger* (E), and *P. guilliermondii* (F) and fresh raw garlic (RAW) on *E. coli* (G), *A. niger* (H), and *P. guilliermondii* (I). The results are represented as means ± SD of triplicate independent readings. Values carried with different letters show significantly different (*p* < 0.05).

Garlic fermented in longan honey (FGH) exhibited IZ values in the control group similar to those of FGE and FGV, measuring 23.0 ± 0.0, 12.3 ± 0.6, and 12.0 ± 1.0 mm, respectively for *E. coli* , *A. niger* , and *P. guilliermondii*. Figure 2A shows that the highest IZ for *E. coli* was recorded at the end of week 1 (26.7 ± 1.5 mm), followed by a gradual decrease, reaching a minimum of 10.7 ± 0.6 mm at 2 months. For *A. niger* (Figure 2B), peak IZ values were observed during weeks 1 and 2 at 25.0 ± 0.0 mm, followed by a decline to the lowest level at week 4 (11.0 ± 0.0 mm). From month 2 onward, IZ values exhibited slight fluctuations before increasing in months 3 and 5. In the case of *P. guilliermondii* (Figure 2C), the highest IZ was recorded at the end of month 2 (21.7 ± 0.6 mm), while the lowest IZ occurred at week 2. An initial IZ increase was observed at month 2 (11.7 ± 0.6 mm), followed by a decline at the sixth month. Table S3 demonstrates the antimicrobial activity of garlic fermented in honey, alongside MIC values. The MIC for *E. coli* was 4 mg/L at weeks 1 and 2, *A. niger exhibited an* increase from 5 to 9 mg/L, and *P. guilliermondii* showed a MIC of 16 mg/L at the end of the second month.

AGE60 contains abundant water‐soluble compounds, such as SAC and SAMC, which are the primary sulfur‐containing components in aged garlic. The extraction process selectively favors water‐soluble bioactive compounds, such as phenols, flavonoids, pyruvate, and thiosulfate. Garlic aged at 60°C and 95% RH exhibited superior antimicrobial activity compared to other samples. Control measurements for AGE60 yielded IZ values of 23.0 ± 0.0, 13.0 ± 1.7, and 12.0 ± 1.0 mm, respectively, for *E. coli* , *A. niger* , and *P. guilliermondii*. As shown in Figure 2D, *E. coli* exhibited the highest IZ (16.8 ± 1.0 mm) at week 1 and the lowest (11.7 ± 0.6 mm) at the end of the fourth month. Similarly, *A. niger* displayed antimicrobial activity akin to *E. coli* , with an IZ of 17.7 ± 0.6 mm in week 1 and the lowest (11.3 ± 0.6 mm) in week 4 (Figure 2E). After the fourth week, IZ values were no longer observable, suggesting a significant reduction in AGE6's efficacy against mold growth. Regarding *P. guilliermondii*, Figure 2E demonstrates that the highest IZ of 24.3 ± 1.2 mm occurred in week 2, while the lowest (12.3 ± 0.6 mm) was observed in weeks 1 and 3, as well as in the fifth month. Table S4 provides a summary of IZ and MIC values for AGE60. The MIC for *E. coli* was determined to be 6 mg/L in the third month of the experiment. For *A. niger* , an MIC of 100 mg/L was observed from months 2 to 5, while *P. guilliermondii* exhibited its lowest MIC value of 9 mg/L at month 5. Discrepancies between expected IZ and MIC values suggest that experimental limitations may have influenced the results.

Fresh RAW garlic samples were not subjected to any processing treatment. Allicin, a well‐documented antimicrobial compound, is abundantly present in RAW garlic. Control experiments subjected to aqueous extracts of fresh RAW garlic yielded IZ values of 23.0 ± 0.0, 12.7 ± 1.2, and 12.3 ± 1.5 mm for *E. coli* , *A. niger*, and *P. guilliermondii*, respectively. As demonstrated in Figure 2G, *E. coli* exhibited a maximum IZ of 26.7 ± 1.5 mm at the end of week 1, whereas the lowest IZ was recorded at 14.0 ± 0.0 mm in week 4. Following this point in the experiment, IZ values gradually and slightly increased by the end of month six. A clear pattern in IZ values was observed, with an initial decline after week one, reaching the lowest point in week 4, followed by a modest increase through month 6. For *A. niger* (Figure 2H), the highest IZ of 25.0 ± 0.0 mm was recorded while the lowest IZ of 14.7 ± 2.1 mm occurred in month two of the fermentation period. After the second month of exposure to RAW garlic, no IZ values were visible. In the case of *P. guilliermondii*, Figure 2I shows that the highest IZ of 27.3 ± 0.6 mm was observed at month four, whereas the lowest IZ of 12.7 ± 1.2 mm was evident in week 3 and month 3. Table S5 summarizes the antimicrobial activity exhibited by fresh raw garlic extracts during the experiment. In addition, the MIC values for RAW garlic did not correspond with IZ values obtained from the agar well diffusion method. The MIC for *E. coli* remained at 6 mg/L from week 1 through month 2, while *A. niger* exhibited a slight increase in MIC to 14 mg/L from months 3 through 6. *P. guilliermondii* demonstrated a substantially higher MIC of 65 mg/L. Correlation analyses between FGE, FGV, FGH, AGE60, and RAW garlic extract values, as shown in Table 1, reveal significant associations among some samples. The antimicrobial activity of AGE in *A. niger* demonstrated a correlation coefficient of 0.975 with FGV in *A. niger* , indicating similar trends in efficacy. RAW garlic extract for *E. coli* exhibited a correlation coefficient of 0.836 with FGH for the same microorganism. Likewise, RAW garlic extract for *A. niger* displayed correlation coefficients of 0.842 and 0.873 with FGV and AGE60 respectively.

**TABLE 1:** Correlation analysis among FGE, FGV, FGH, AGE60, and RAW on inhibition zone (IZ) of *E. coli, A. niger*, and *P. guilliermondii*.

Correlation analysis among FGE, FGV, FGH, AGE60, and RAW values on IZ of *E. coli* (Ec), *A. niger* (An), and *P. guilliermondii* (Pg)															
*R* ^2^ value	FGE Ec	FGE An	FGE Pg	FGV Ec	FGV An	FGV Pg	FGH Ec	FGH An	FGH Pg	AGE Ec	AGE An	AGE Pg	RAW Ec	RAW An	RAW Pg
FGE Ec	1	−0.524^a^	0.101	0.463^b^	0.727^a^	0.069	0.607^a^	−0.068	−0.358	0.486^a^	0.770^a^	0.088	0.571^a^	0.559^a^	−0.226
FGE An		1	0.445^b^	−0.089	−0.171	−0.107	−0.434^b^	0.478^a^	0.383^b^	−0.377^b^	−0.272	−0.073	−0.177	−0.332	0.344
FGE Pg			1	0.237	0.432^b^	0.415^b^	0.137	0.244	0.169	−0.269	0.316	−0.223	0.048	0.194	−0.282
FGV Ec				1	0.436^b^	0.001	0.509^a^	−0.071	0.075	0.694^a^	0.388^b^	−0.501^a^	0.524^a^	0.130	−0.179
FGV An					1	0.218	0.663^a^	0.225	−0.152	0.064	0.975^a^	−0.043	0.633^a^	0.842^a^	−0.287
FGV Pg						1	0.448^b^	0.283	−0.275	−0.358	0.117	−0.175	0.278	−0.011	−0.069
FGH Ec							1	0.287	−0.369^b^	0.327	0.642^a^	0.086	0.836^a^	0.391^b^	−0.287
FGH An								1	−0.105	−0.314	0.117	0.369^b^	0.537^a^	−0.096	0.257
FGH Pg									1	−0.196	−0.277	−0.073	−0.182	−0.034	0.037
AGED Ec										1	0.140	−0.211	0.311	−0.104	−0.082
AGED An											1	−0.023	0.576^a^	0.873^a^	−0.312
AGED Pg												1	0.106	0.028	0.033
RAW Ec													1	0.284	0.099
RAW An														1	−0.381^b^
RAW Pg															1

^a^ Correlation is significant at the 0.01 level (2‐tailed).

^b^ Correlation is significant at the 0.05 level (2‐tailed).

Garlic exhibits a broad spectrum of antibacterial and antifungal properties. The antifungal activity of garlic extract is primarily attributed to allicin, a bioactive compound present in fresh garlic extract. The inhibition of fungal growth observed in this study may be related to allicin, which alters the function of enzymes essential for fungal metabolism. Its primary mechanism likely involves penetration of the cell wall and organelles, leading to the destruction of the cellular structure, cytoplasm leakage, and modulation of important macromolecules (Kuna et al. 2022; Li et al. 2014). The media used for garlic fermentation and pickling serve as effective aseptic environments. Ethanol functions as an ideal sterile medium, fruit vinegar maintains a low pH, and honey has been extensively documented in scientific literature for its antimicrobial properties. Therefore, the observed antimicrobial effects may be attributed not only to allicin but also to a synergistic interaction between allicin and antimicrobial compounds present in ethanol, vinegar, and honey. In the case of raw garlic, allicin serves as the main antibacterial agent, compromising bacterial cell structure and disrupting metabolic processes (Shang et al. 2019). The findings of this study align with previous research (Olivas‐Mendez et al. 2022), which indicates that raw garlic extracts exhibit stronger antimicrobial activity than processed garlic extracts. Consequently, raw garlic was included in the comparative study, demonstrating the highest inhibitory effect on *E. coli* , *A. niger* , and *P. guilliermondii* using the agar well diffusion method. Moreover, several studies have selected the same pathogenic bacteria and employed similar assessment methods as those utilized in this investigation (Ahmad Nejhad et al. 2023; Bergen et al. 2024).

**FIGURE 3:**
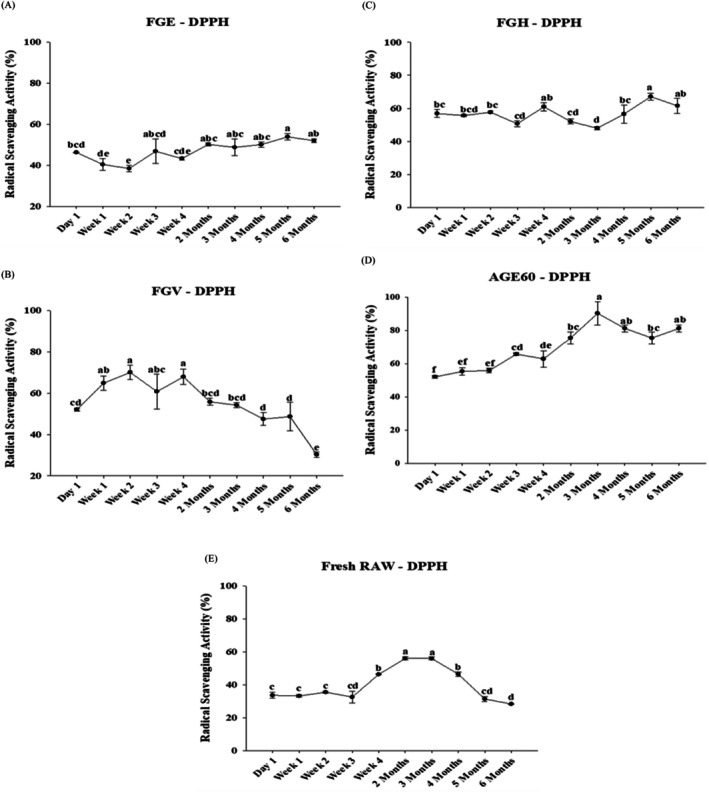
The free radical scavenging activity (DPPH) analysis of different processing methods of garlic has been detected within 6 months. The garlic fermented in 95% ethanol‐FGE (A), garlic fermented in fruit vinegar‐FGV (B), garlic fermented in honey‐FGH (C), garlic fermented in aged black garlic‐AGE60 (D), and fresh garlic‐RAW (E), expressed as percentage (%). The results are represented as means ± SD of triplicate independent readings. Values carried with different letters show significantly different (*p* < 0.05).

Figure 3A illustrates the radical scavenging activity (RSA) of garlic fermented in 95% ethanol (FGE). On day 1, FGE exhibited an RSA of 46.3% ± 0.2%, which subsequently decreased to 38.4% ± 1.5% by week two, ranking as the third‐lowest overall. However, by month five, FGE demonstrated its highest RSA value of 53.9% ± 1.6%, yet it remained the lowest among all garlic samples. Similarly, Figure 3B presents the RSA of garlic fermented in fruit vinegar (FGV), which initially recorded an RSA of 52.0% ± 0.8% on day 1. By the end of the sixth month, FGV exhibited the lowest RSA within its category at 30.3% ± 1.2%, ranking as the fourth lowest overall. Notably, the highest RSA for FGV was observed in week two, 70.0% ± 3.6%, making it the second‐highest overall. Garlic fermented in honey (FGH), shown in Figure 3C, exhibited a relatively high RSA of 67.1% ± 2.2% in the fifth month, ranking third overall. The lowest RSA for FGH occurred in the third month at 48.0% ± 1.0%, which was lower than its initial RSA of 56.9% ± 2.5% recorded on day 1. This initial RSA ranked as the second‐lowest overall among all samples. Figure 3D highlights the RSA of aged black garlic at 60°C (AGE60), which demonstrated the highest RSA among all samples, reaching 90.3% ± 7.1% on the third month. Conversely, AGED60 exhibited the lowest RSA on day 1, with a value of 52.0% ± 0.8%, which ranked first in terms of the lowest RSA compared to other garlic samples. Lastly, Figure 3E shows the RSA of fresh raw garlic (RAW), which ranked as the second‐lowest overall, with its highest RSA reaching 55.9% ± 0.9%. Within its category, RAW exhibited its lowest RSA at 28.2% ± 0.3%. Table S6 summarizes the RSA percentage values across different processing times. The displayed values correspond exclusively to the RSA percentage relative to the absorbance of the tested samples and controls. Additionally, establishing a standard curve with an appropriate reference agent would be beneficial for obtaining more precise RSA values.

**FIGURE 4:**
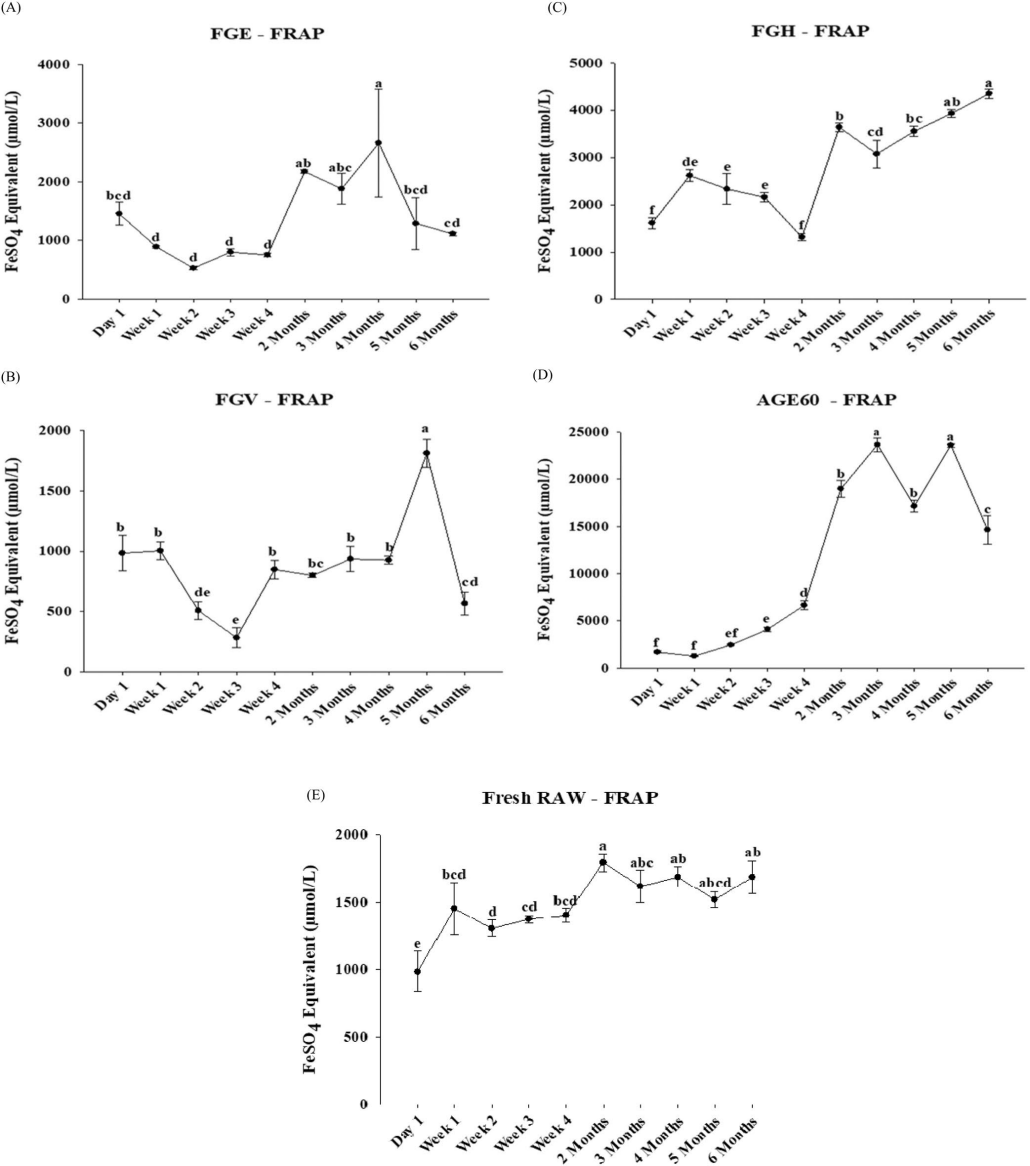
The ferric reducing antioxidant power (FRAP) analysis of different processing methods of garlic has been detected within 6 months. The garlic fermented in 95% ethanol‐FGE (A), garlic fermented in fruit vinegar‐FGV (B), garlic fermented in honey‐FGH (C), garlic fermented in aged black garlic‐AGE60 (D), and fresh garlic‐RAW (E) expressed in μmol/L. The results are represented as means ± SD of triplicate independent readings. Values carried with different letters show significantly different (*p* < 0.05).

The FRAP assay was employed to assess the ferric‐reducing power of garlic extracts. FRAP is a colorimetric assay that operates through a redox‐linked complex in the presence of antioxidant compounds. This assay functions by reducing ferric ions (Fe^3+^) to ferrous ions (Fe^2+^) under acidic conditions, resulting in the formation of a blue‐colored ferrous‐probe complex (Hsu et al. 2024). Figure 4A shows the ferric‐reducing ability of garlic fermented in 95% ethanol (FGE). On day 1, FGE exhibited a ferric‐reducing ability of 1452.2 ± 194.7 μmol FeSO_4_ equivalent/L. The highest ferric‐reducing ability was recorded in month four at 2660.8 ± 919.1 μmol FeSO_4_ equivalent/L, ranking as the third‐highest among all samples. On the other hand, the lowest ferric‐reducing ability of FGE was observed in week two at 526.3 ± 29.3 μmol FeSO_4_ equivalent/L. Similarly, Figure 4B depicts the ferric‐reducing activity of garlic fermented in fruit vinegar (FGV). FGV exhibited the fourth‐highest reducing ability in the fifth month, reaching 1812.9 ± 117.0 μmol FeSO_4_ equivalent/L, while the lowest value was recorded in week three at 282.6 ± 84.4 μmol FeSO_4_ equivalent/L. Fermented garlic in honey (FGH), presented in Figure 4C, exhibited its highest reducing ability at the end of the 6‐month processing period, achieving 4356.7 ± 101.3 μmol FeSO_4_ equivalent/L, ranking as the second‐highest among all samples. The lowest ferric‐reducing activity for FGH was detected in week four of the fermentation period, measuring 1315.8 ± 77.4 μmol FeSO_4_ equivalent/L. Figure 4D shows that aged black garlic processed at 60°C (AGE60) exhibited the highest reducing ability among all garlic samples. The maximum activity was observed in months three and five, with values of 23674.5 ± 751.4 and 23616.0 ± 194.7 μmol FeSO_4_ equivalent/L, respectively. The lowest reducing ability was recorded in week one at 1267.0 ± 60.9 μmol FeSO_4_ equivalent/L, reflecting an initial decline from day 1 (1686.2 ± 118.2 μmol FeSO_4_ equivalent/L), followed by a subsequent increase until the end of the aging process. Figure 4E shows the ferric‐reducing ability of fresh raw garlic (RAW). The highest reducing ability of RAW was recorded at 1793.4 ± 67.5 μmol FeSO_4_ equivalent/L, representing an increase from its initial value of 984.4 ± 150.1 μmol FeSO_4_ equivalent/L. The lowest reducing ability of RAW matched its day 1 value and ranked fifth, making it the lowest among all the garlic samples tested using the FRAP assay. Table S7 provides a comprehensive summary of the ferric‐reducing antioxidant power of different garlic extracts across various processing periods, as determined by the FRAP assay.

**FIGURE 5:**
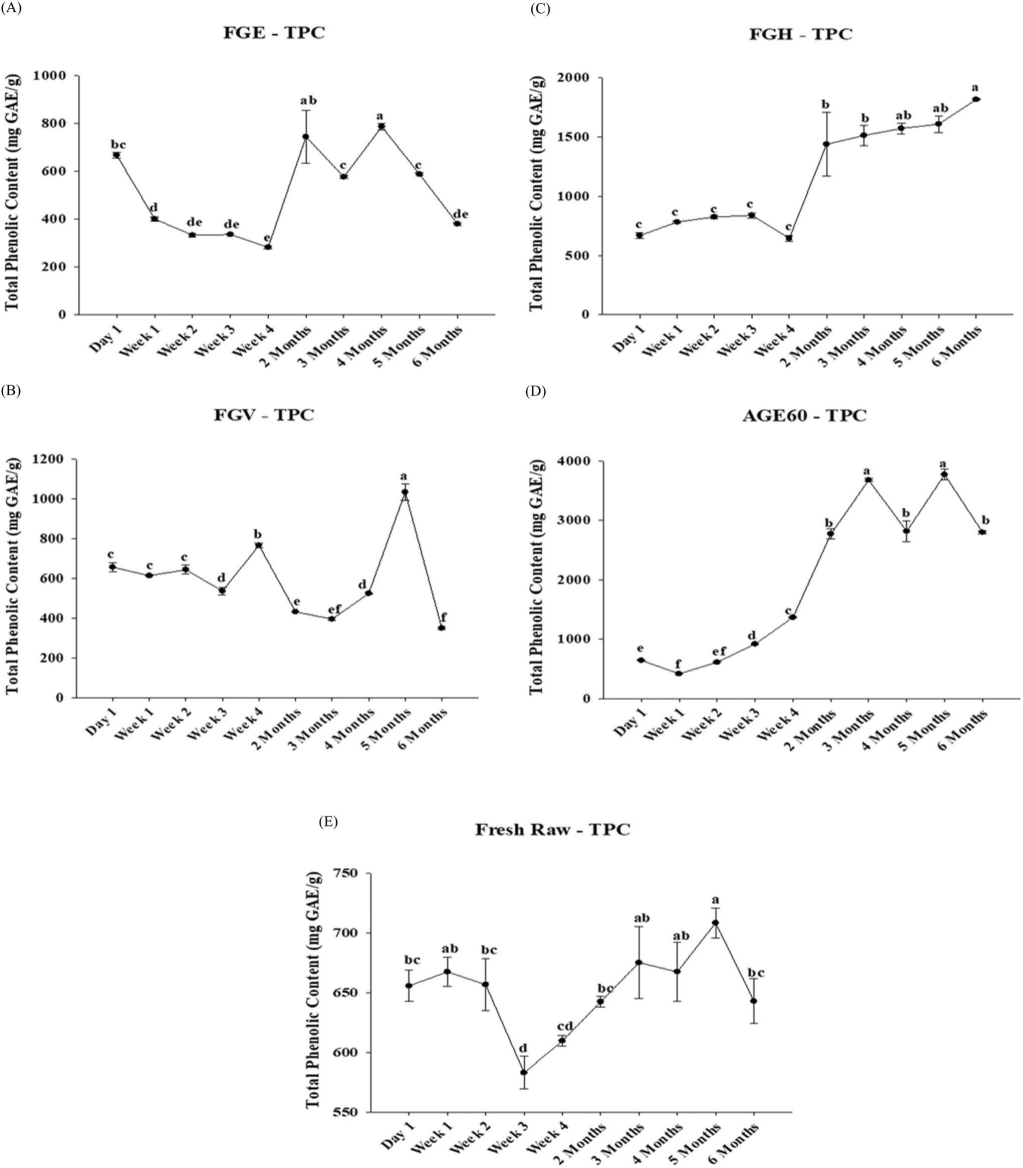
Comparative antioxidant activity of different garlic extracts via total phenolic content (TPC) assay. Total phenolic content of five garlic extracts FGE (A), FGV (B), FGH (C), AGE60 (D), and RAW (E), expressed in mg GAE/g. Bars with different superscripts indicate statistically significant differences among tested samples, analyzed using one‐way ANOVA followed by Tukey's test (*p ≤* 0.05). The total phenolic content (TPC) analysis of different processing methods of garlic has been detected within 6 months. The garlic fermented in 95% ethanol‐FGE (A), garlic fermented in fruit vinegar‐FGV (B), garlic fermented in honey‐FGH (C), garlic fermented in aged black garlic‐AGED60 (D), and fresh garlic‐RAW (E), expressed in mg GAE/g. The results are represented as means ± SD of triplicate independent readings. Values carried with different letters show significantly different (*p* < 0.05).

Figure 5 shows the total phenolic content (TPC) of the garlic samples tested at different experiment time points, and Figure 5A shows the TPC of garlic fermented in 95% ethanol (FGE) as mg GAE/g of the sample. On day 1, FGE exhibited a TPC of 667.5 ± 12.2 mg GAE/g, which decreased to 281.4 ± 5.5 mg GAE/g by week four of the processing period. The highest TPC of FGE was observed in month four, with a TPC of 786.5 ± 12.9 mg GAE/g, ranking fourth among the tested samples. Similarly, Figure 5B shows the TPC of garlic fermented in fruit vinegar (FGV). On day 1, FGV had a TPC of 656.8 ± 21.6 mg GAE/g, which increased approximately 1.5‐fold to 1033.7 ± 40.5 mg GAE/g in the fifth month, ranking third overall. The lowest TPC for FGV was observed in month six, measuring 349.6 ± 5.4 mg GAE/g. Figure 5C illustrates the TPC of garlic fermented in honey (FGH). The highest TPC for FGH was observed in the sixth month at 1815.8 ± 6.2 mg GAE/g, ranking second among all samples and increasing nearly 3‐fold from the initial processing stage. The lowest TPC for FGH occurred at week four, measuring 645.0 ± 24.5 mg GAE/g, reflecting a slight decrease from its day 1 value of 667.5 ± 24.7 mg GAE/g. Figure 5D displays the TPC of garlic aged at 60°C (AGE60). AGE60 exhibited the highest levels of TPC among all samples in months three and five at 3679.3 ± 24.7 and 3771.6 ± 89.4 mg GAE/g, respectively. From an initial TPC of 642.4 ± 4.7 mg GAE/g on day 1, the TPC increased almost six‐fold. The lowest TPC for AGE60 was observed at the end of week one, measuring 416.3 ± 9.0 mg GAE/g. Previous studies stated that thermal treatment combined with high relative humidity significantly enhances antioxidant activity by the third month of processing (Zhang et al. 2015), which is consistent with the findings of this experiment. Figure 5E illustrates the TRPC of fresh raw garlic (RAW). The highest TPC for RAW was observed in the fifth month at 708.5 ± 12.4 mg GAE/g, following a slight increase from its day one value of 655.7 ± 13.2 mg GAE/g. RAW ranked the lowest among all samples. The lowest TPC for RAW was observed at the end of week three, measuring 582.9 ± 13.7 mg GAE/g. Line graph points with distinct superscripts indicate significant differences between samples at *p* ≤ 0.05. Table S8 summarizes the variations in TPC across different garlic samples at different extraction time points during the experimental processing period. The predominant antioxidant compounds identified in this study were total phenolic compounds. By the end of the processing period, AGE60 showed the highest level of free radical scavenging activity, followed sequentially by honey, ethanol, raw garlic, and vinegar.

Table 2 illustrates the correlation between the total phenolic content (TPC) of garlic extracts and their antioxidant activity, as measured using the ferric reducing antioxidant power (FRAP) assay. The TPC of FGE exhibited a strong correlation with FRAP, with a coefficient of 0.843. Similarly, the phenolic content of FGH demonstrated a relationship with AGE60 in terms of radical scavenging activity (DPPH), FRAP, and TPC, with correlation coefficients of 0.844, 0.922, and 0.860, respectively. Furthermore, AGE60 exhibited strong correlations with FGH for TPC (0.889), DPPH (0.894), and FRAP (0.990). The observed increase in the antioxidant activity of garlic extracts suggests that the processing method plays a vital role in influencing the synthesis and retention of antioxidant compounds. Among the tested samples, AGE60, FGH, and FGV demonstrated the most promising antioxidant properties across different assays, including DPPH, FRAP, and TPC. AGE60 exhibited the highest FRAP and TPC values over extended processing periods, while FGH and FGV also presented elevated levels of polyphenols, likely due to the inherent bioactive compounds in fruit vinegar and honey. These findings support the hypothesis that sugars contribute to the formation of specific antioxidant compounds, like Amadori and Heynes products, during the Maillard reaction, which varies depending on the presence of reducing sugars and organic acids. Previous studies have suggested that raw garlic possesses stronger antioxidant activity than those processed by fermentation; however, the results of this study do not support that conclusion. Instead, AGE60 garlic extract exhibited the highest antioxidant activity among all tested samples. The high phenolic content of garlic, as observed in these experiments, may be responsible for its enhanced antioxidant properties.

**TABLE 2:** Correlation analysis among FGE, FGV, FGH, AGE60, and RAW extracts on DPPH, FRAP, and TPC values.

Correlation analysis among FGE, FGV, FGH, AGE60, and RAW on DPPH, FRAP, and TPC values															
*R* ^2^ value	DPPHFGE	DPPHFGV	DPPHFGH	DPPHAGE	DPPHRAW	FRAPFGE	FRAPFGV	FRAPFGH	FRAPAGE	FRAPRAW	TPCFGE	TPCFGV	TPCFGH	TPCAGE	TPC RAW
DPPHFGE	1	−0.627^a^	0.177	0.671^a^	0.084	0.503^a^	0.339	0.658^a^	0.736^a^	0.485^a^	0.500^a^	−0.044	0.734^a^	0.765^a^	0.221
DPPHFGV		1	−0.243	−0.539^a^	0.190	−0.400^b^	−0.181	−0.691^a^	−0.507^a^	−0.346	−0.353	0.284	−0.745^a^	−0.580^a^	−0.287
DPPHFGH			1	−0.171	−0.498^a^	−0.149	0.495^a^	0.185	0.045	−0.080	−0.127	0.632^a^	0.136	0.108	0.291
DPPHAGE				1	0.448^b^	0.521^a^	0.120	0.687^a^	0.881^a^	0.742^a^	0.368^b^	−0.382^b^	0.844^a^	0.894^a^	0.195
DPPHRAW					1	0.565^a^	0.006	0.010	0.472^a^	0.439^b^	0.444^b^	−0.341	0.174	0.398^b^	0.011
FRAP FGE						1	0.255	0.438^b^	0.592^a^	0.466^a^	0.843^a^	−0.300	0.538^a^	0.559^a^	0.266
FRAP FGV							1	0.268	0.475^a^	0.061	0.411^b^	0.671^a^	0.270	0.468^a^	0.724^a^
FRAP FGH								1	0.734^a^	0.713^a^	0.389^b^	−0.240	0.922^a^	0.752^a^	0.462^b^
FRAPAGE									1	0.695^a^	0.530^a^	−0.069	0.860^a^	0.990^a^	0.450^b^
FRAPRAW										1	0.247	−0.376^b^	0.723^a^	0.669^a^	0.112
TPC FGE											1	−0.102	0.470^a^	0.489^a^	0.445^b^
TPC FGV												1	−0.275	−0.068	0.307
TPC FGH													1	0.889^a^	0.420^b^
TPC AGE														1	0.454^b^
TPC RAW															1

^a^ Correlation is significant at the 0.01 level (2‐tailed).

^b^ Correlation is significant at the 0.05 level (2‐tailed).

## 4 Conclusion

**FIGURE 6:**
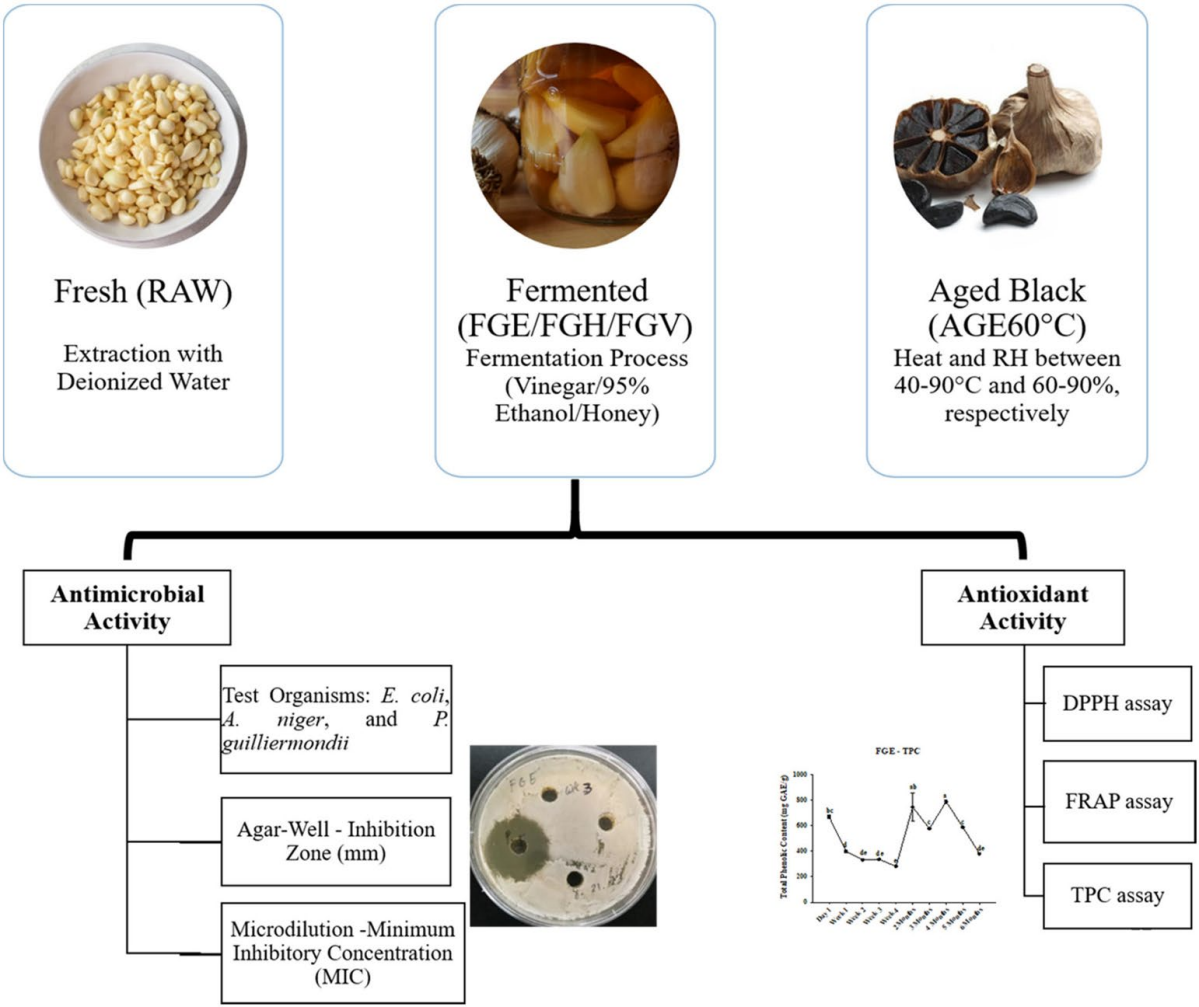
Overview of garlic preparations, classification, and evaluation of antimicrobial and antioxidant activities of different processed garlic samples.

The study evaluated the antimicrobial and antioxidant properties of different processed garlic samples (Figure 6). The results demonstrated that different processing methods influenced the levels of antimicrobial, antibacterial, antifungal, and antioxidant activities. Among the tested samples, FGV, FGH, FGE, and AGE60 exhibited notable antimicrobial activity. However, fresh raw garlic (RAW) displayed the strongest antimicrobial properties, mainly due to its high allicin content and other sulfur‐containing organosulfur compounds (OSCs), which are regarded as potent antimicrobial agents. In terms of antioxidant activity, AGE60, FGH, and FGV proved to be the most effective. The fermentation and aging processes contributed to the production of flavonoids and phenolic compounds, enhancing the antioxidant properties of these garlic samples. However, these processing methods did not significantly improve antimicrobial properties. These findings suggest that different garlic processing methods confer distinct functional benefits, particularly in human health. Therefore, individuals may select a suitable processed garlic product based on their specific health needs, whether for antimicrobial or antioxidant benefits. Further research is warranted to explore the underlying mechanisms of these bioactive compounds and their potential applications in food preservation and therapeutic interventions.

## Author Contributions

**Luis M. Cocom:** data curation (lead), formal analysis (equal). **Hsiao‐Chi Wang:** funding acquisition (equal), investigation (supporting), writing – review and editing (equal). **Kuo‐Chan Tseng:** funding acquisition (equal), supervision (equal). **Yung‐Lin Chu:** conceptualization (lead), funding acquisition (equal), supervision (lead), validation (equal), writing – original draft (lead).

## Conflicts of Interest

The authors declare no conflicts of interest.

## Supporting information


**Table S1:** fsn370743‐sup‐0001‐Tables.docx.

## Data Availability

Data are openly available in a public repository that issues datasets with DOIs.
