# Supplementary material for: The Antimicrobial and Antioxidant Properties of Raw, Aged, and Fermented Garlic: Influence of Processing Methods
Source: Food Sci Nutr. 2025 Jul 31;13(8):e70743. doi: 10.1002/fsn3.70743 (PMC12311394; doi:10.1002/fsn3.70743)
Supplement: Supplementary file 1 — Table S1: fsn370743‐sup‐0001‐Tables.docx. [file FSN3-13-e70743-s001.docx]

**Supplementary data**

***Sup Table 1.* Inhibition zones of *E. coli, A. niger*, and *P. guilliermondii* treated with FGE.**

| **Fermented Garlic in 95% Ethanol (FGE) – Antimicrobial Susceptibility Testing** | | | | |
| --- | --- | --- | --- | --- |
| Week 1 | Week 2 | Week 3 | Week 4 | 2 Months |
| *E. coli* | | | | |
| 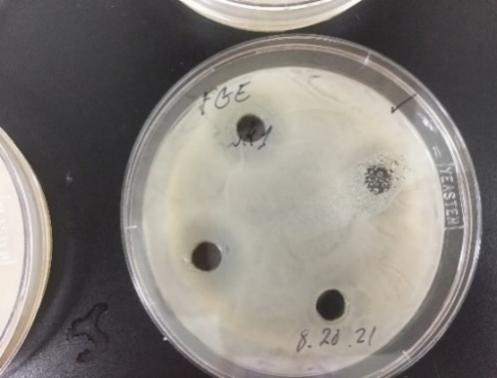 | 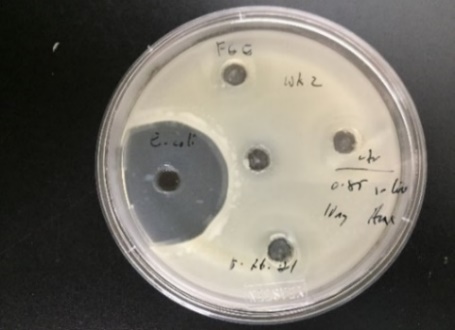 | *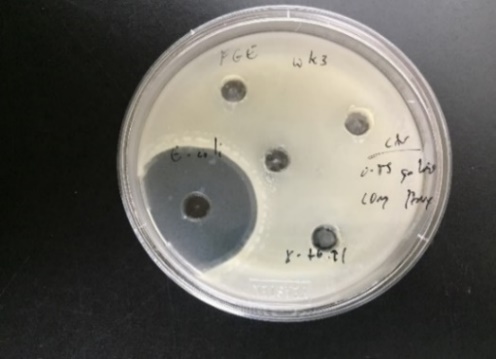* | 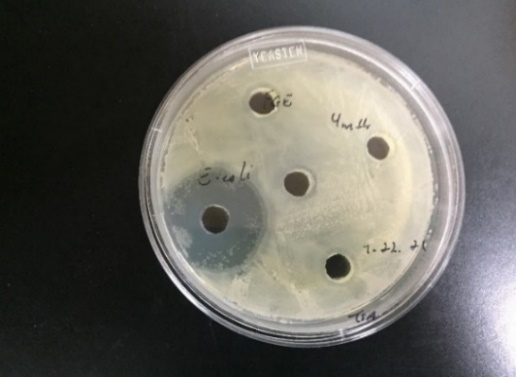 | 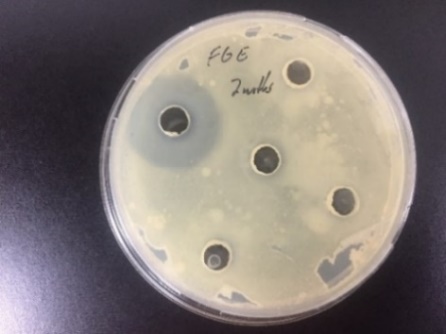 |
| *A. niger* | | | | |
| 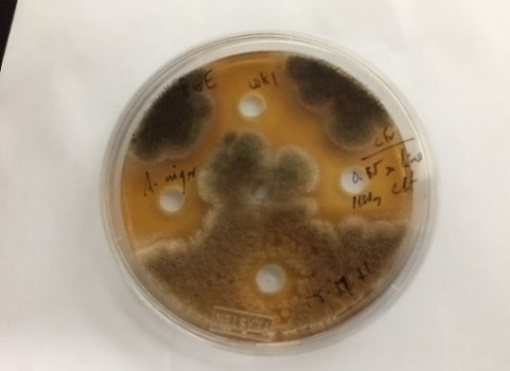 | 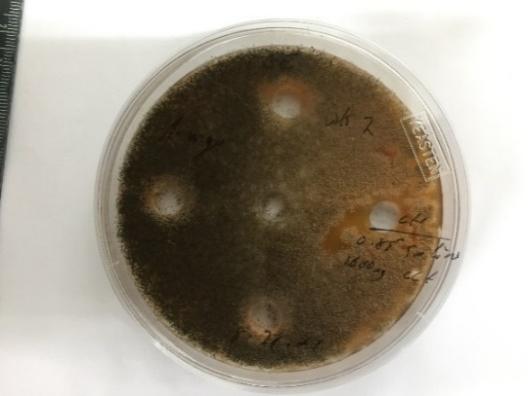 | *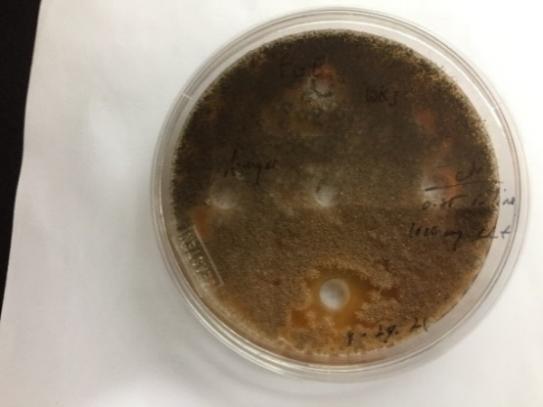* | 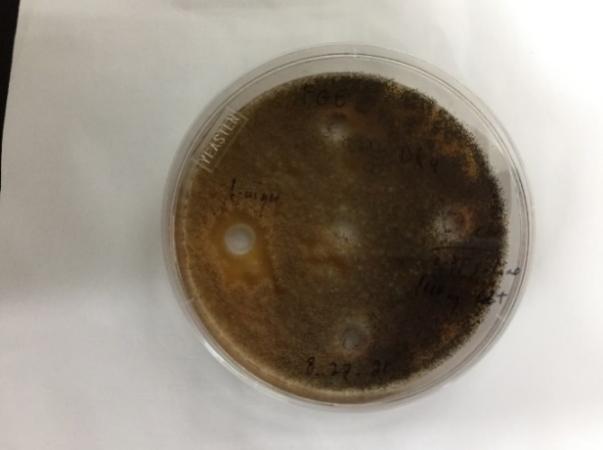 | 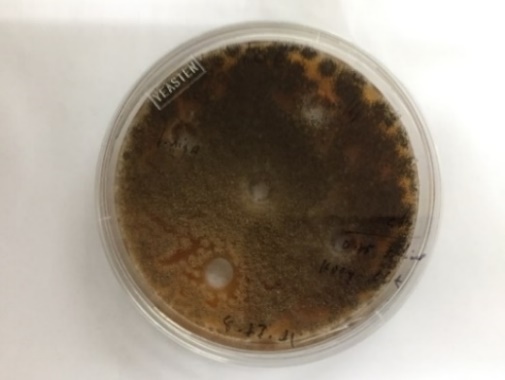 |
| *P. guilliermondii* | | | | |
| 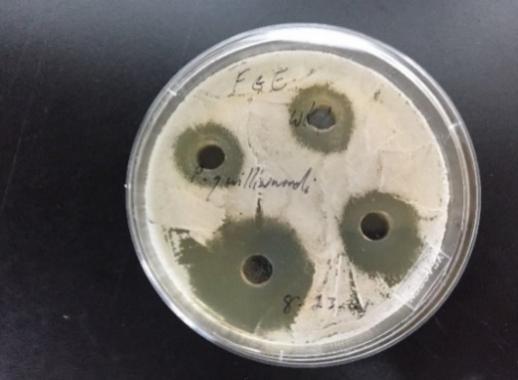 | 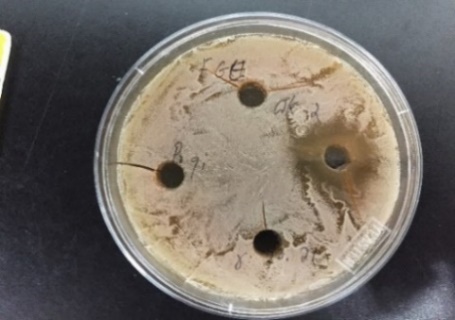 | 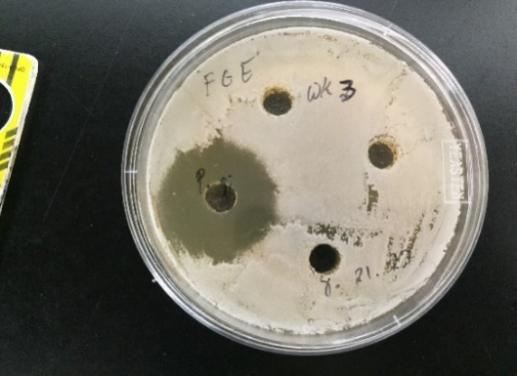 | 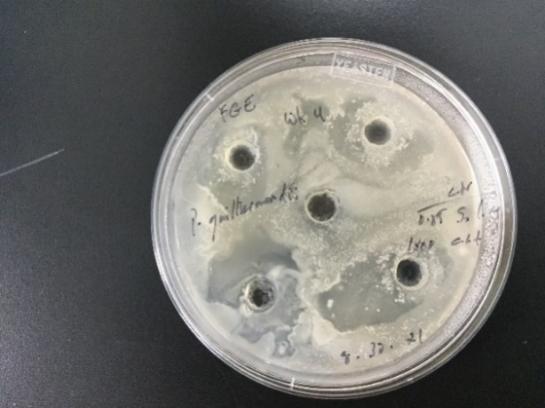 | 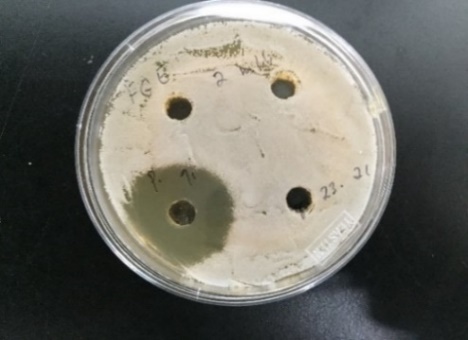 |
| 3 Months | 4 Months | 5 Months | 6 Months |  |
| *E. coli* | | | |  |
| 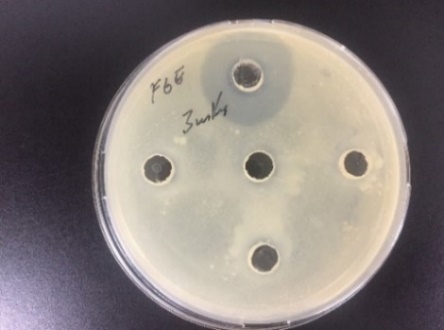 | 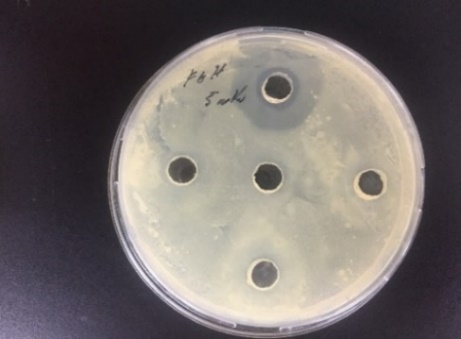 | *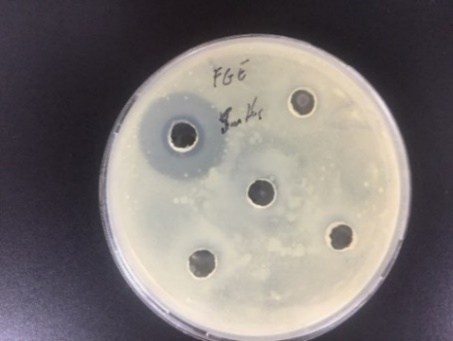* | N/A |  |
| *A. niger* | | | |  |
|  |  |  |  |  |
| *P. guilliermondii* | | | |  |
| 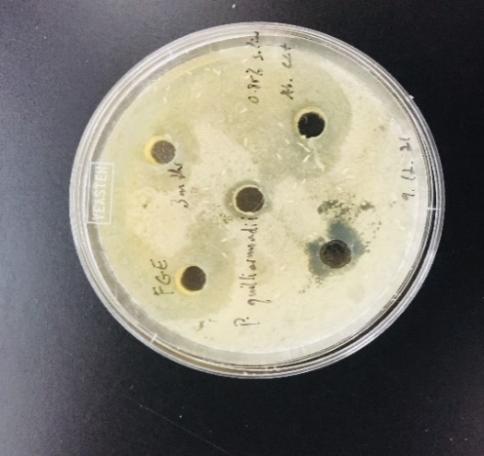 | 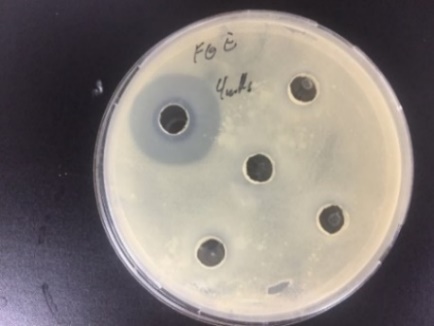 | 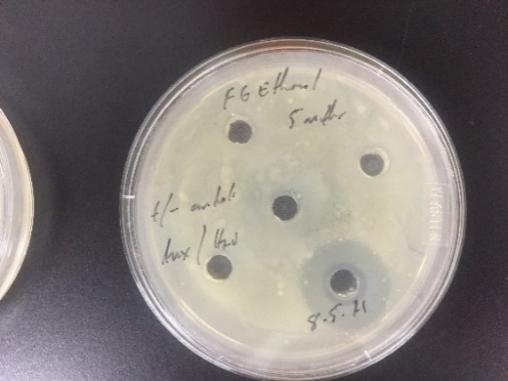 | N/A |  |

**Sup Table 2. Inhibition zones of E. coli, A. niger, and P. guilliermondii treated with FGV.**

| **Fermented Garlic in Fruit Vinegar (FGV) – Antimicrobial Susceptibility Testing** | | | | |
| --- | --- | --- | --- | --- |
| Week 1 | Week 2 | Week 3 | Week 4 | 2 Months |
| *E. coli* | | | | |
| N/A | N/A | *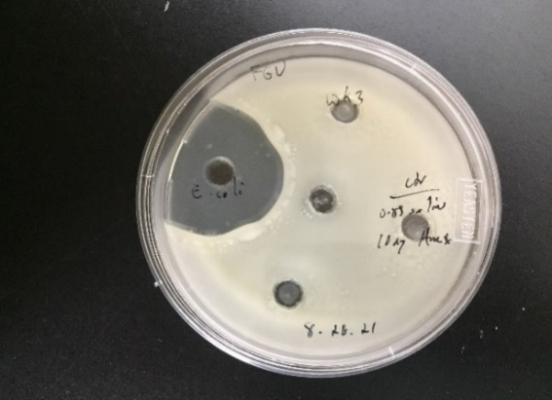* | N/A | 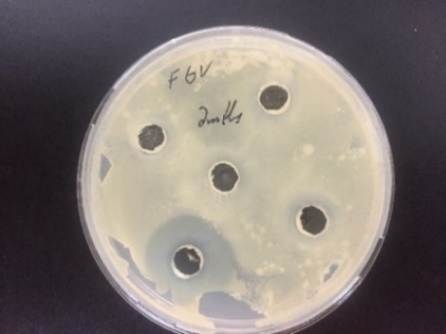 |
| *A. niger* | | | | |
| 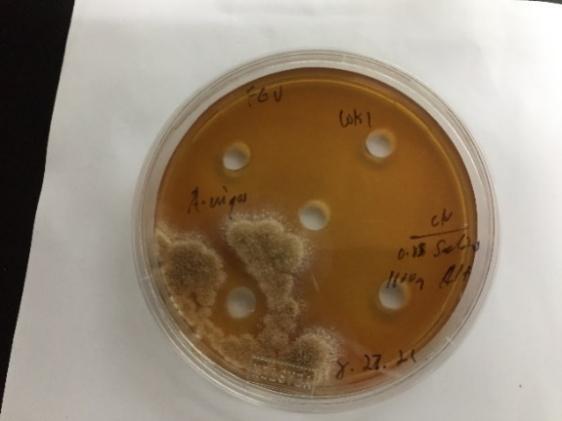 | 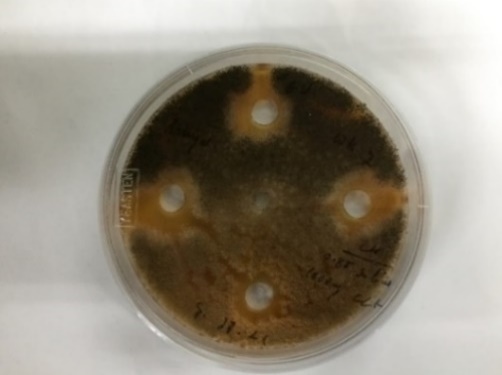 | *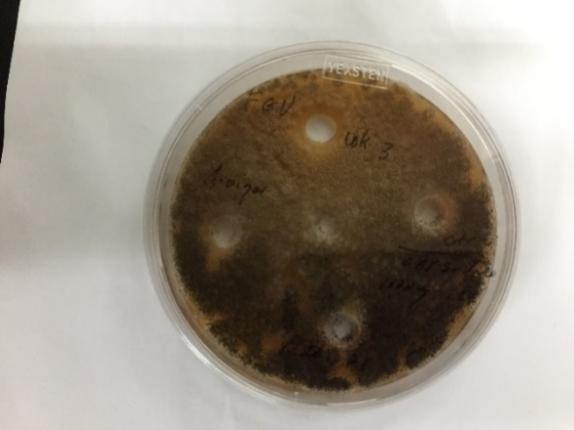* | 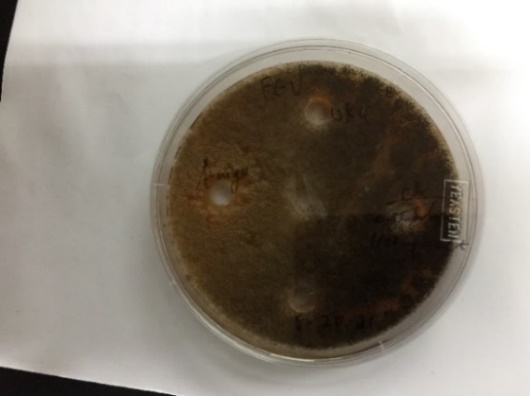 | 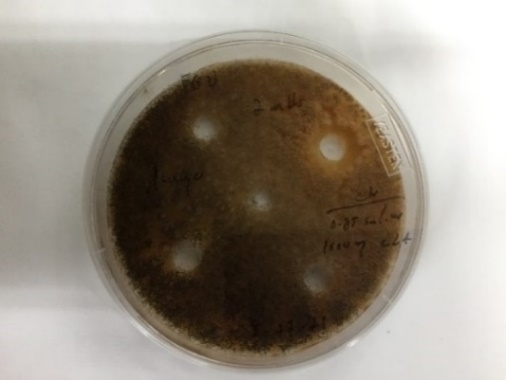 |
| *P. guilliermondii* | | | | |
| 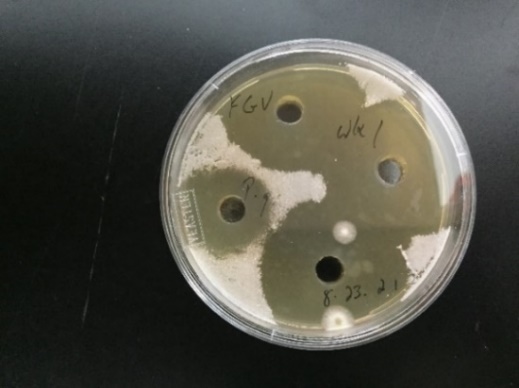 | 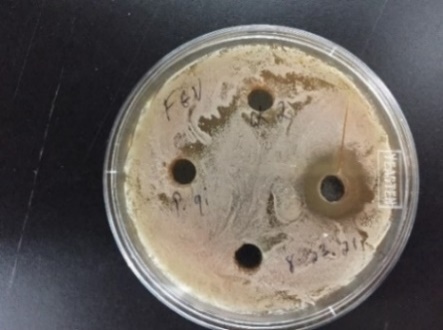 | *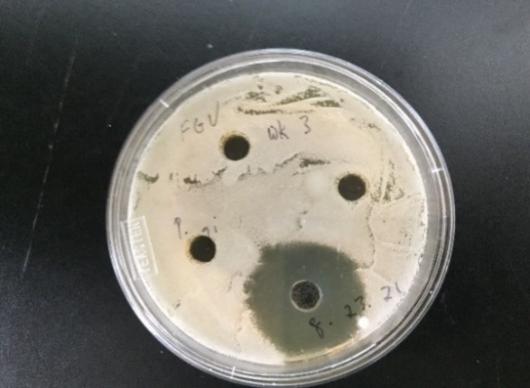* | 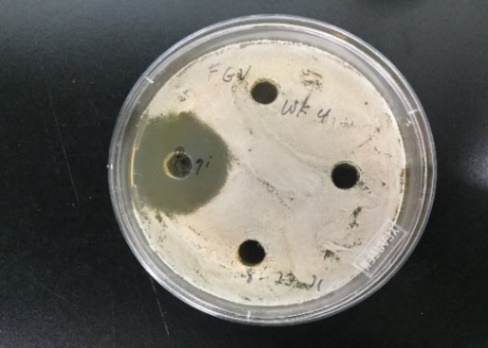 | 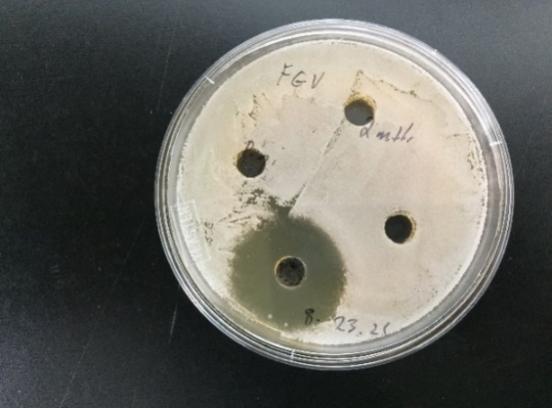 |
| 3 Months | 4 Months | 5 Months | 6 Months |  |
| *E. coli* | | | |  |
| 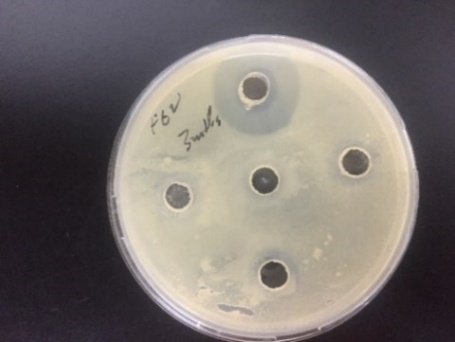 | 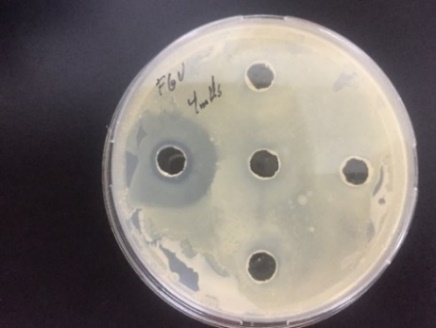 | *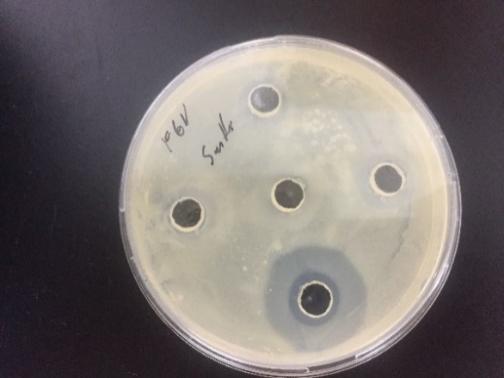* | N/A |  |
| *A. niger* | | | |  |
| N/A | N/A | N/A | N/A |  |
| *P. guilliermondii* | | | |  |
| N/A | N/A | N/A | N/A |  |

**Sup** **Table 3. Inhibition zones (IZ) of E. coli, A. niger, and P. guilliermondii treated with FGH.**

| **Fermented Garlic in Honey (FGH) – Antimicrobial Susceptibility Testing** | | | | |
| --- | --- | --- | --- | --- |
| Week 1 | Week 2 | Week 3 | Week 4 | 2 Months |
| *E. coli* | | | | |
| 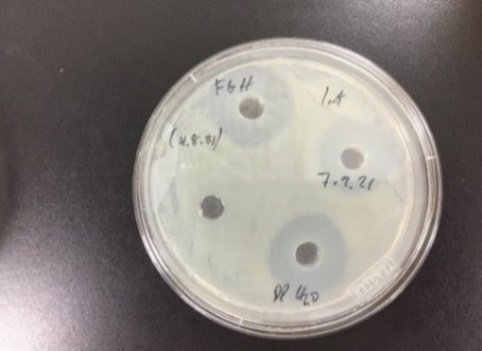 | 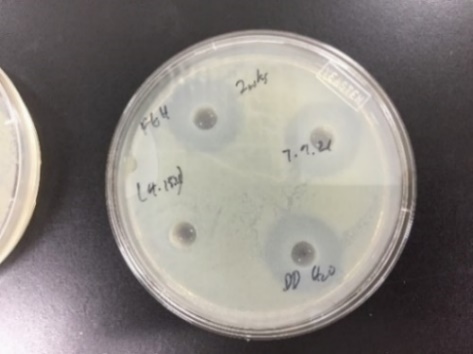 | *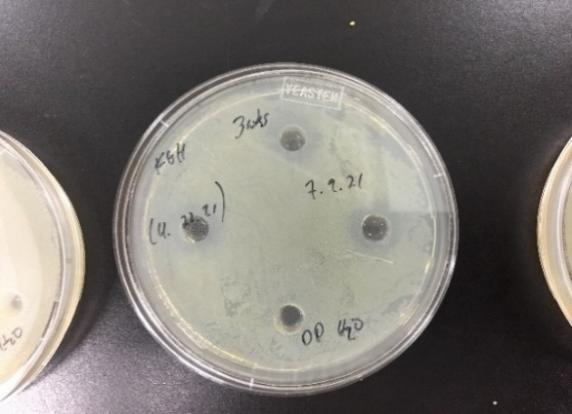* | 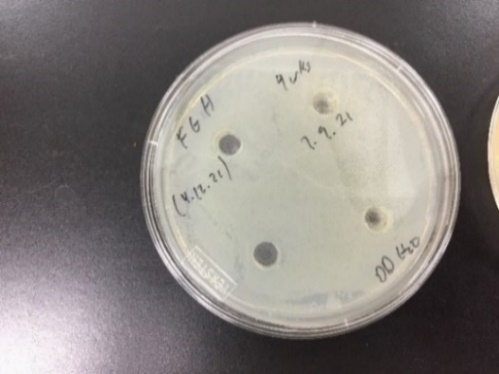 | 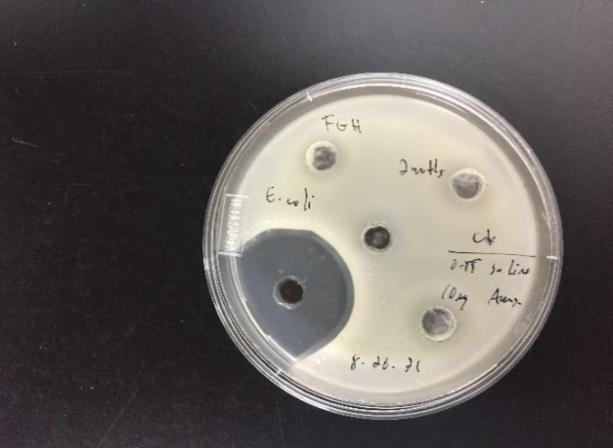 |
| *A. niger* | | | | |
| 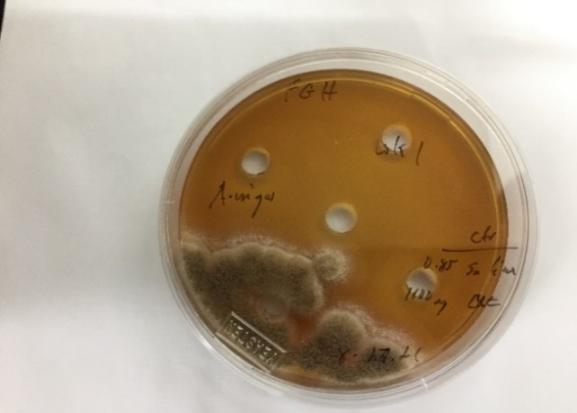 | 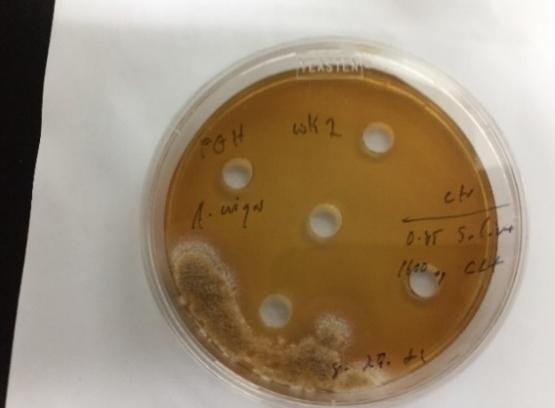 | *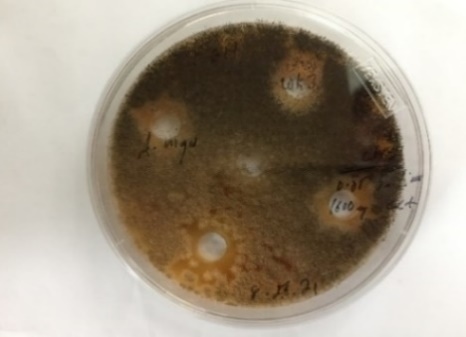* | 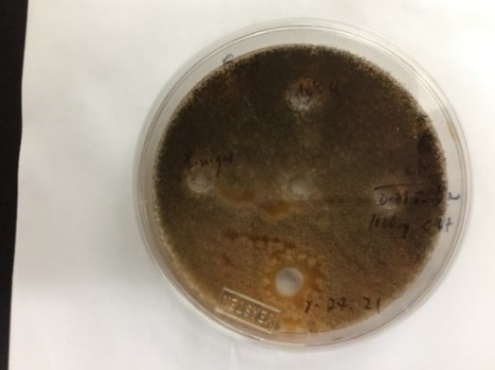 | 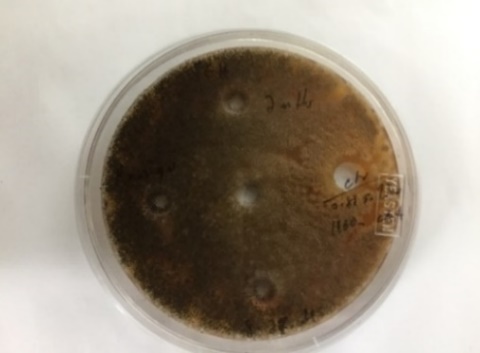 |
| *P. guilliermondii* | | | | |
| 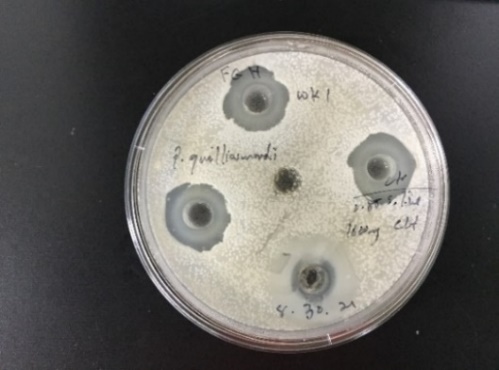 | 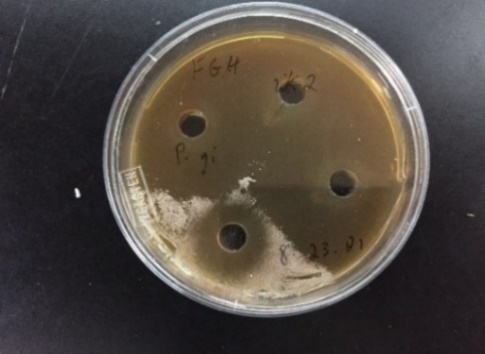 | 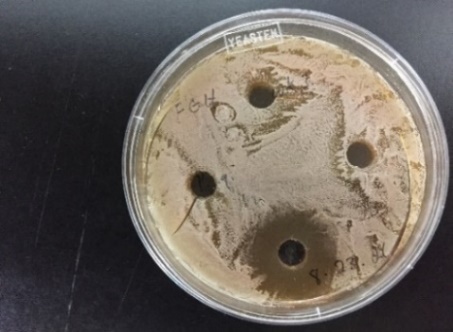 | 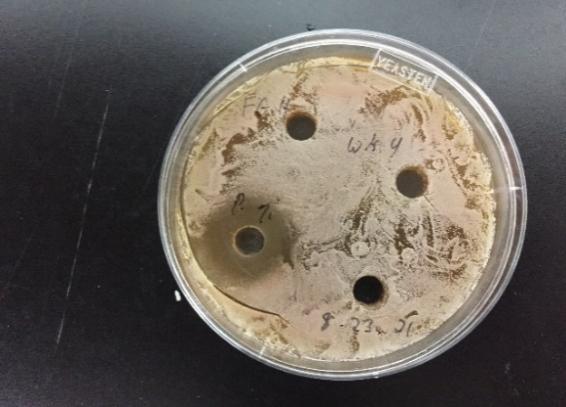 | 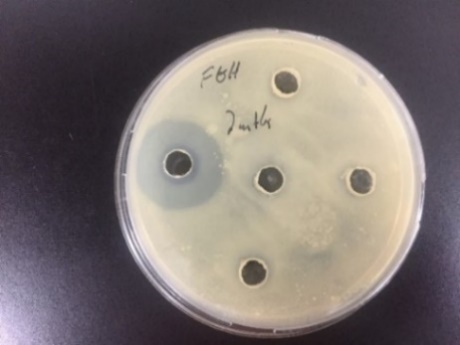 |
| 3 Months | 4 Months | 5 Months | 6 Months |  |
| *E. coli* | | | |  |
| 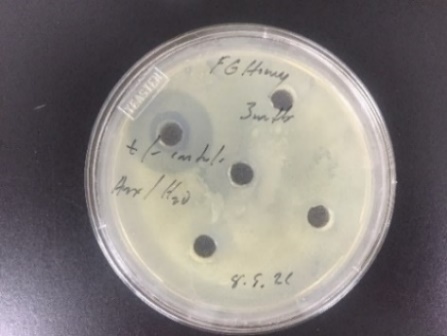 | 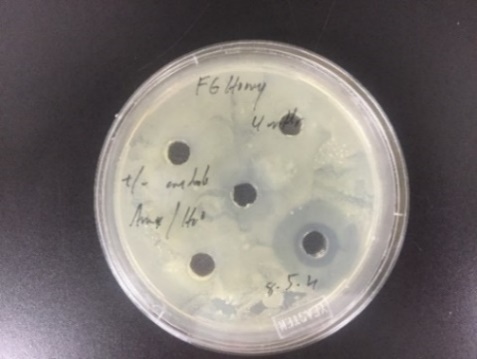 | *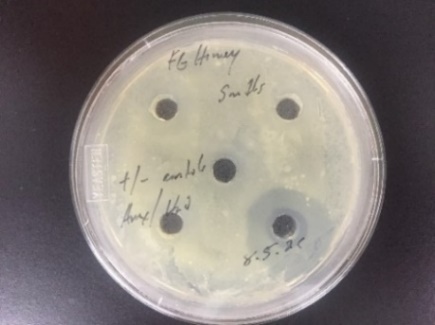* | N/A |  |
| *A. niger* | | | |  |
| N/A | N/A | N/A | N/A |  |
| *P. guilliermondii* | | | |  |
| 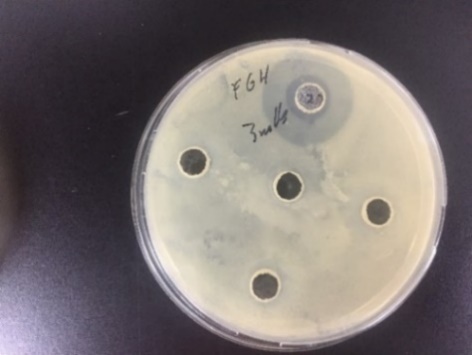 | 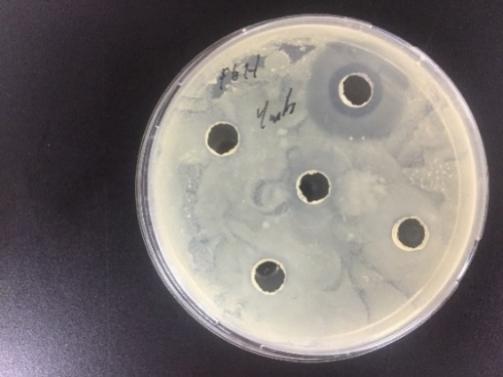 | *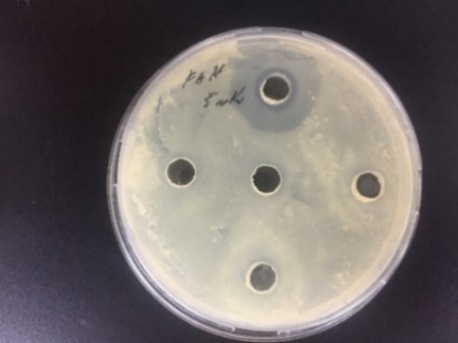* | N/A |  |

**Sup** **Table 4. Inhibition zones of E. coli, A. niger, and P. guilliermondii treated with AGED60.**

| **Aged Garlic at 60°C – Antimicrobial Susceptibility Testing** | | | | |
| --- | --- | --- | --- | --- |
| Week 1 | Week 2 | Week 3 | Week 4 | 2 Months |
| *E. coli* | | | | |
| 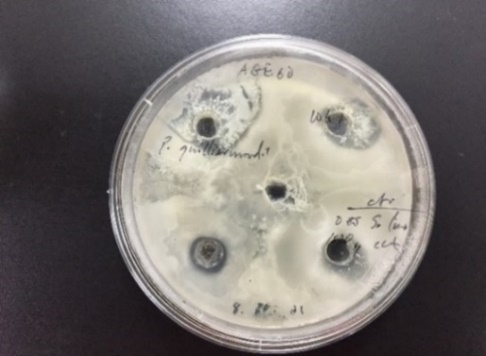 | 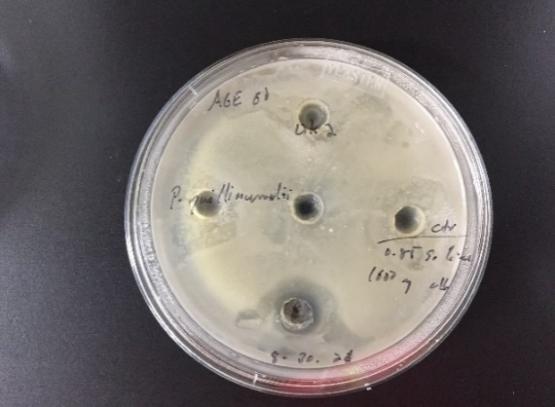 | N/A | 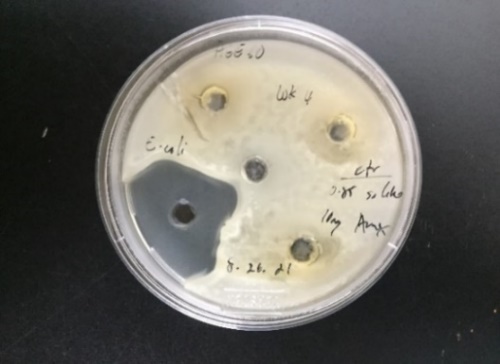 | 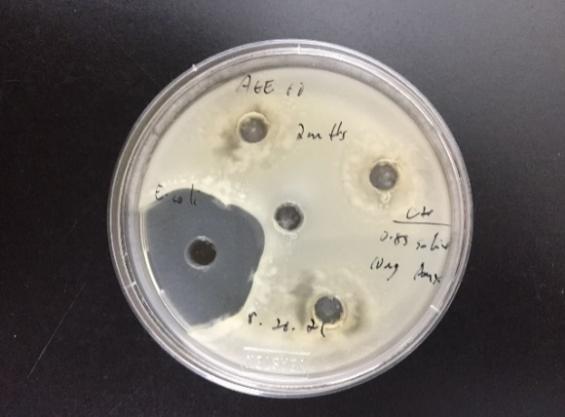 |
| *A. niger* | | | | |
| 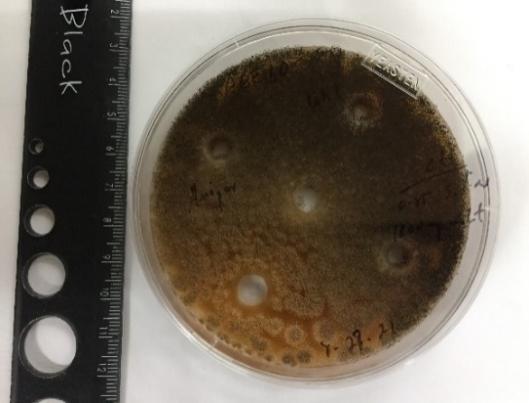 | 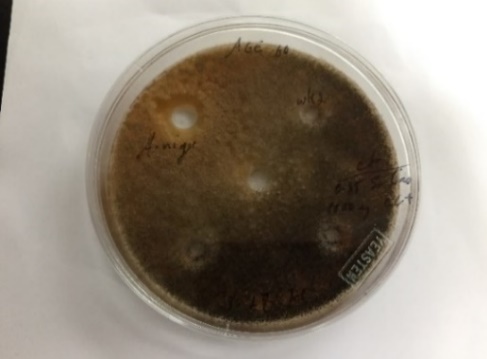 | *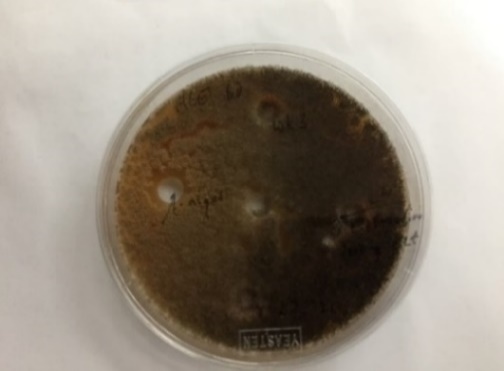* | 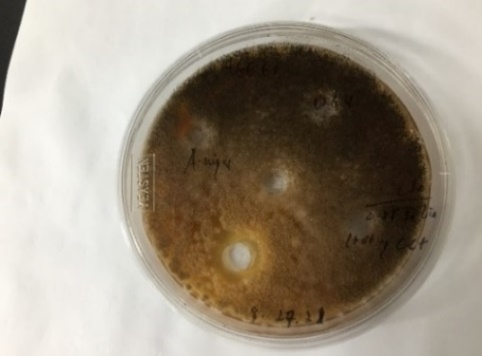 | 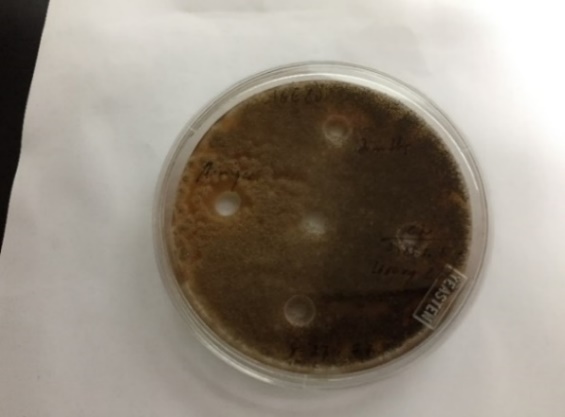 |
| *P. guilliermondii* | | | | |
| 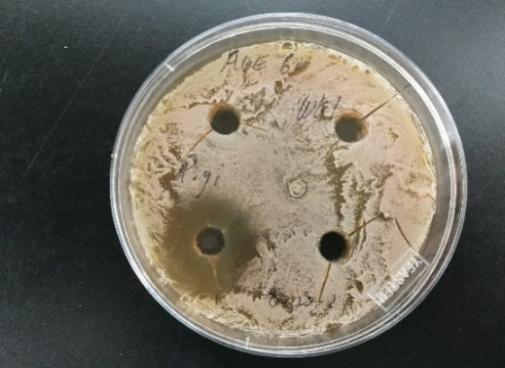 | 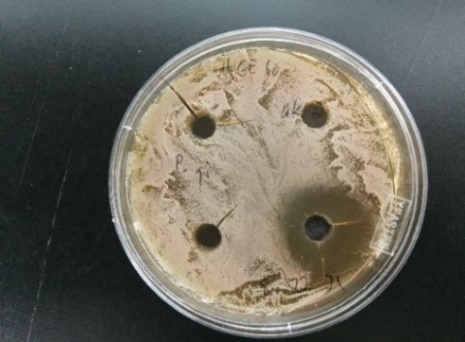 | 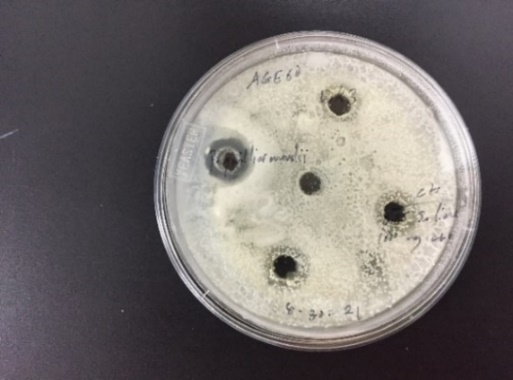 | 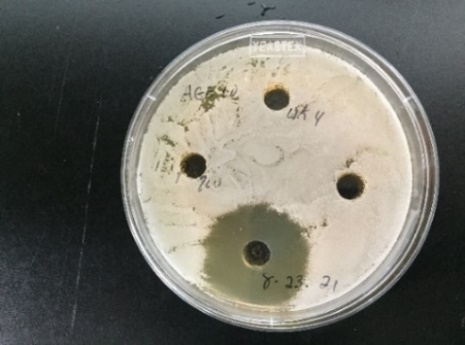 | 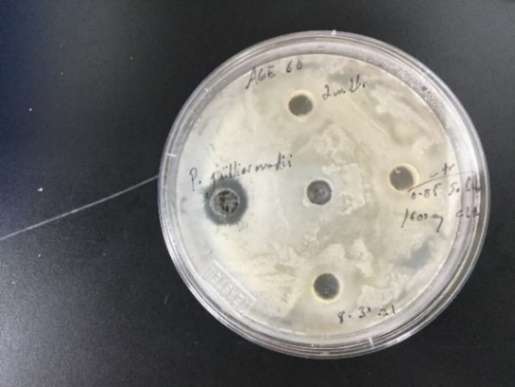 |
| 3 Months | 4 Months | 5 Months | 6 Months |  |
| *E. coli* | | | |  |
| 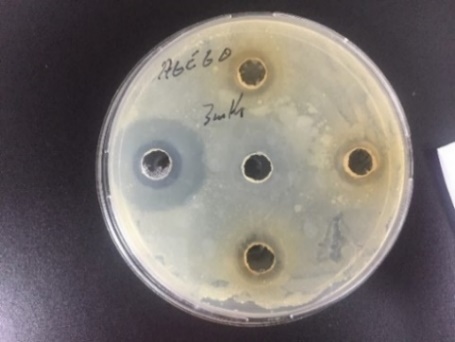 | 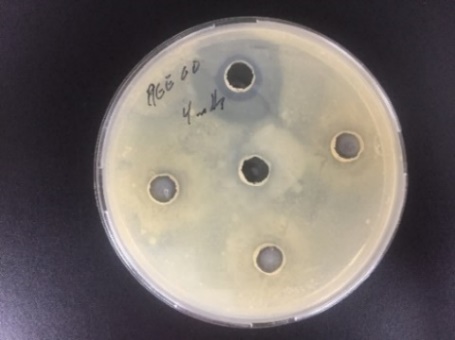 | 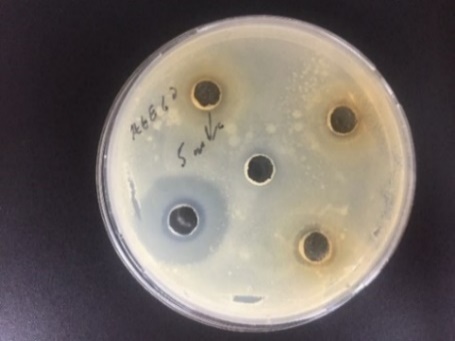 | 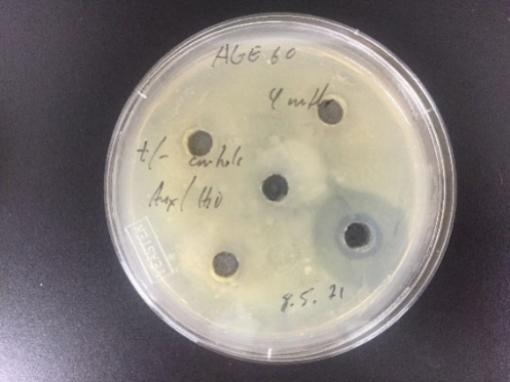 |  |
| *A. niger* | | | |  |
| N/A | N/A | N/A | N/A |  |
| *P. guilliermondii* | | | |  |
| 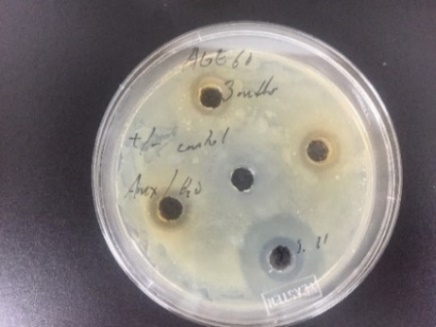 | 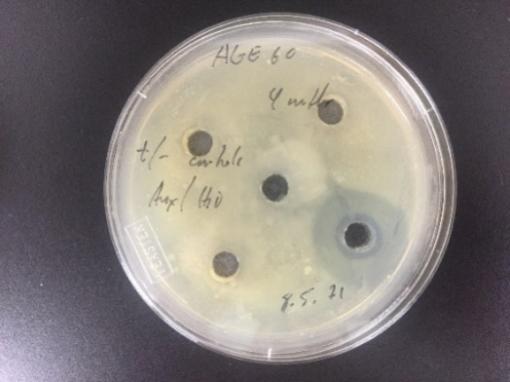 | N/A | 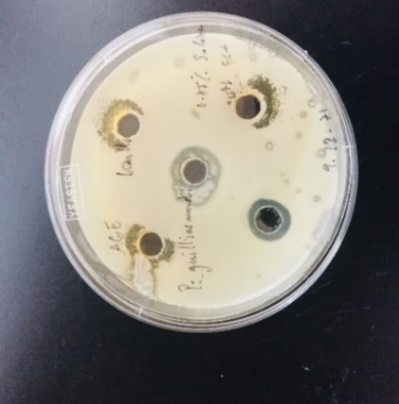 |  |

**Sup** **Table 5. Inhibition zones of E. coli, A. niger, and P. guilliermondii treated with RAW.**

| **Fresh Raw Garlic – Antimicrobial Susceptibility Testing** | | | | |
| --- | --- | --- | --- | --- |
| Week 1 | Week 2 | Week 3 | Week 4 | 2 Months |
| *E. coli* | | | | |
| 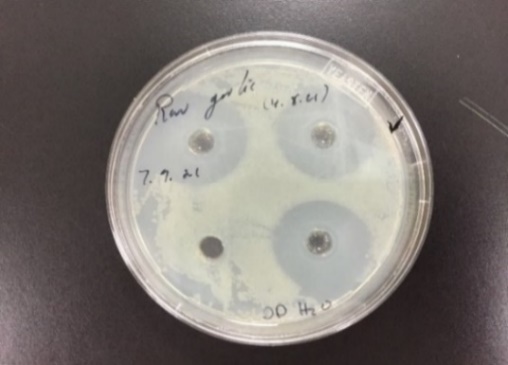 | 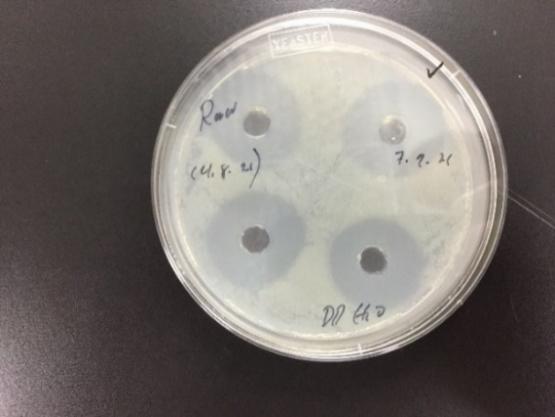 | N/A | N/A | N/A |
| *A. niger* | | | | |
| 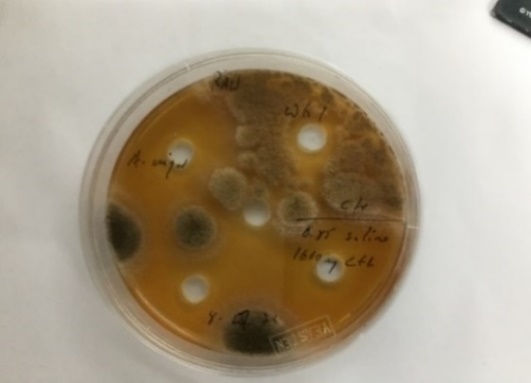 | 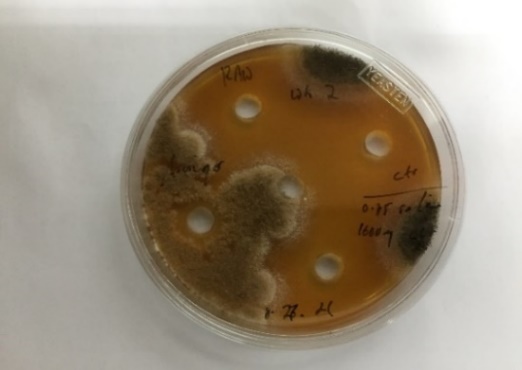 | *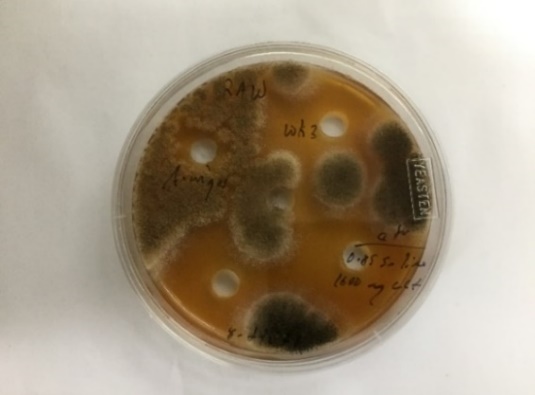* | 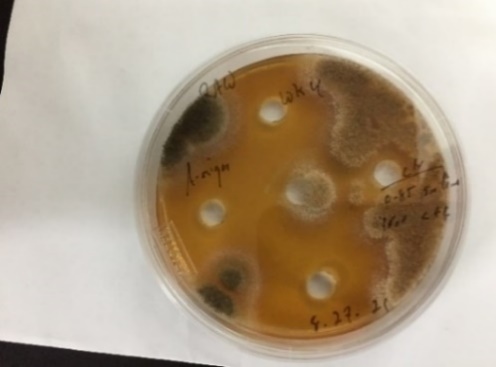 | 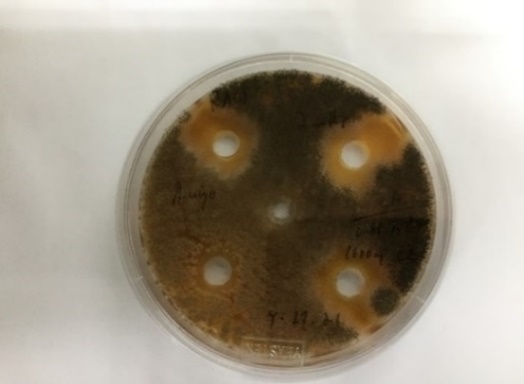 |
| *P. guilliermondii* | | | | |
| 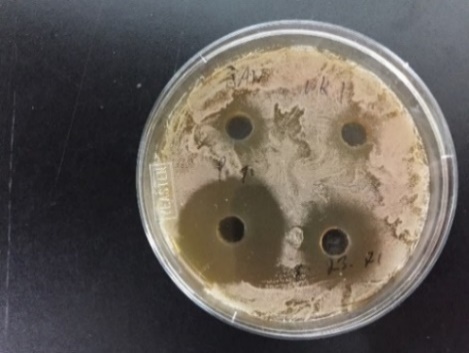 | 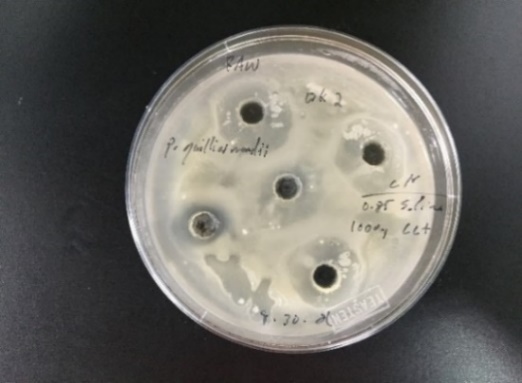 | 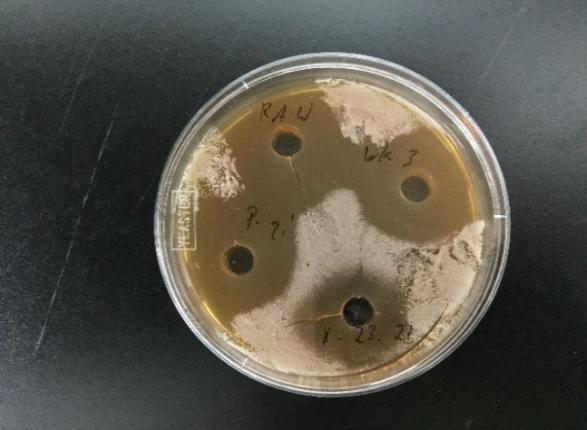 | 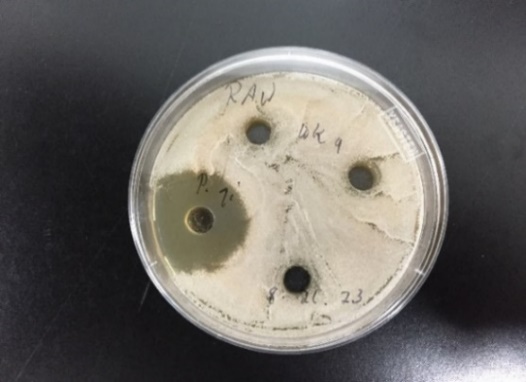 | 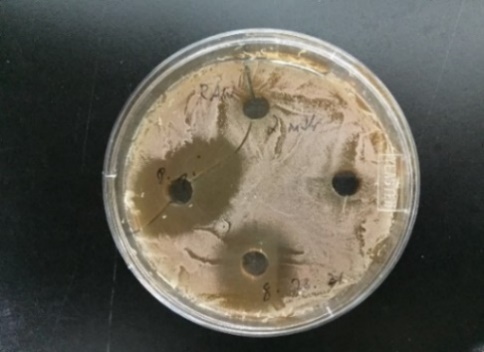 |
| 3 Months | 4 Months | 5 Months | 6 Months |  |
| *E. coli* | | | |  |
| 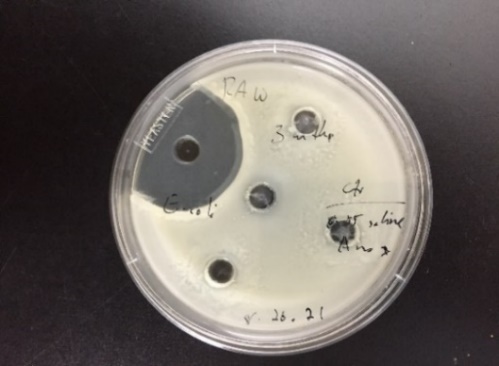 | 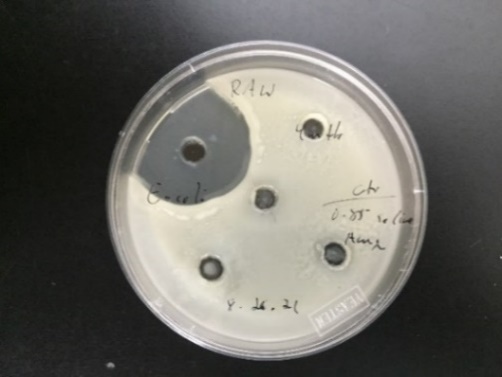 | N/A | 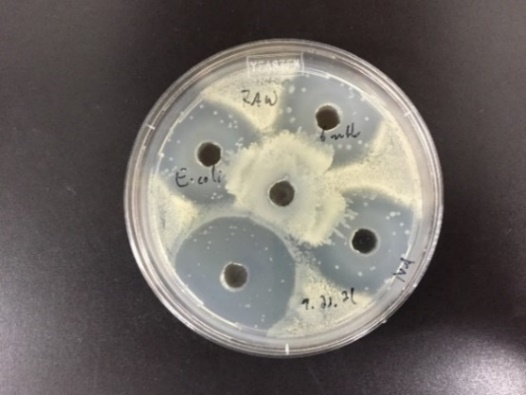 |  |
| *A. niger* | | | |  |
| N/A | N/A | N/A | N/A |  |
| *P. guilliermondii* | | | |  |
| N/A | 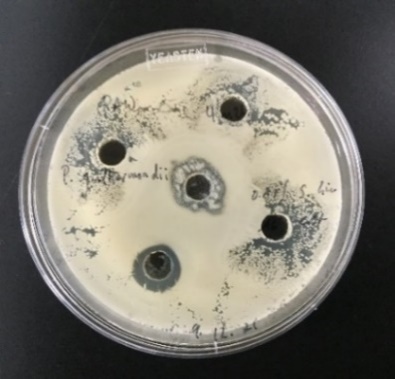 | 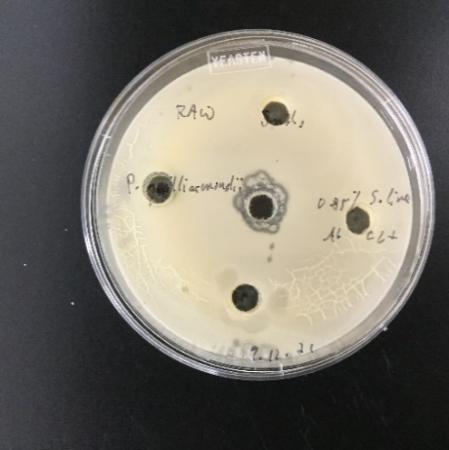 | 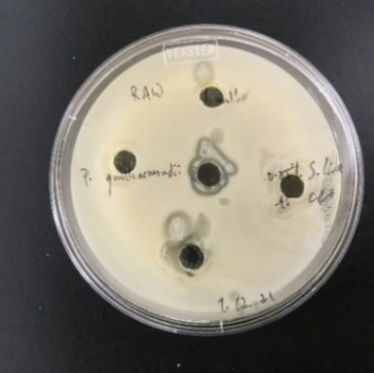 |  |

**Sup Table 6****. Free radical scavenging activity (%) of different processed garlic extracts. Antioxidant activity (DPPH) of processed garlic extracts as a percentage of free radical scavenging activity.**

| **Free Radical Scavenging Activity (%) of Garlic Products - DPPH** | | | | | | | | | | |
| --- | --- | --- | --- | --- | --- | --- | --- | --- | --- | --- |
| **Garlic Sample** | **Processing Period** | | | | | | | | | |
|  | **Day 1** | **Week 1** | **Week 2** | **Week 3** | **Week 4** | **2 Months** | **3 Months** | **4 Months** | **5 Months** | **6 Months** |
| **FGE** | 46.3±0.2^bcd^ | 40.4±3.0^dc^ | 38.4±1.5^e^ | 46.8±6.0^abcd^ | 43.3±0.6^cde^ | 50.1±0.7^abc^ | 48.7±4.0^abc^ | 50.1±1.2^abc^ | 53.9±1.6^a^ | 52.0±0.8^ab^ |
| **FGV** | 52.0±0.8^cd^ | 64.8±3.5^ab^ | 70.0±3.6^a^ | 60.8±8.5^abc^ | 67.9±3.7^a^ | 55.8±1.7^bcd^ | 54.1±1.2^bcd^ | 47.5±3.1^d^ | 48.6±6.9^d^ | 30.3±1.2^e^ |
| **FGH** | 56.9±2.5^bc^ | 55.6±0.6^bcd^ | 57.6±0.7^bc^ | 50.7±1.8^cd^ | 61.0±2.5^ab^ | 52.0±1.5^cd^ | 48.0±1.0^d^ | 56.6±5.5^bc^ | 67.1±2.2^a^ | 61.5±4.6^ab^ |
| **AGE60** | 52.0±0.8^f^ | 55.3±2.2^ef^ | 55.9±1.3^ef^ | 65.7±0.7^cd^ | 62.9±4.9^de^ | 75.4±3.6^bc^ | 90.3±7.1^a^ | 81.2±2.1^ab^ | 75.4±3.6^bc^ | 81.2±2.1^ab^ |
| **RAW** | 33.6±1.8^c^ | 33.2±0.8^c^ | 35.4±0.7^c^ | 32.5±3.6^cd^ | 46.3±0.2^b^ | 55.9±0.9^a^ | 55.9±0.9^a^ | 46.4±1.4^b^ | 31.3±1.6^cd^ | 28.2±0.3^d^ |

Values are expressed as mean ± SD (n=3). Means with different superscripts within rows are significantly different when analyzed by One-way ANOVA, and the Tukey test at *P ≤ 0.05*. Fermented garlic in Ethanol (FGE), fermented garlic in vinegar (FGV), fermented garlic in honey (FGH), aged garlic at 60°C/95% RH (AGED60), and fresh raw garlic (RAW) extracts.

**Sup Table 7. Ferric reducing antioxidant power (FRAP - FeSO_4_ µmol/L) of garlic products of ferrous sulfate equivalent as micromole per liter.**

| **Ferric Reducing Antioxidant Power (FRAP) of Processed Garlic Products - FeSO4 Equivalent (µmol/L)** | | | | | | | | | | |
| --- | --- | --- | --- | --- | --- | --- | --- | --- | --- | --- |
| **Garlic Sample** | **Processing Period** | | | | | | | | | |
|  | **Day 1** | **Week 1** | **Week 2** | **Week 3** | **Week 4** | **2 Months** | **3 Months** | **4 Months** | **5 Months** | **6 Months** |
| **FGE** | 1452.2±194.7^bcd^ | 886.9±16.9^d^ | 526.3±29.3^d^ | 799.2±60.9^d^ | 750.5±33.8^d^ | 2173.5±33.8^ab^ | 1881.1±265.3^abc^ | 2660.8±919.1^a^ | 1286.5±441.5^bcd^ | 1111.1±29.3^cd^ |
| **FGV** | 984.4±150.1^b^ | 1003.9±73.6^b^ | 506.8±73.6^de^ | 282.6±84.4^e^ | 847.9±77.4^b^ | 799.2±16.7^bc^ | 935.7±105.4^b^ | 925.9±33.8^b^ | 1812.9±117.0^a^ | 565.3±94.0^cd^ |
| **FGH** | 1617.9±118.2^f^ | 2621.8±121.7^de^ | 2339.2±325.6^e^ | 2163.7±105.4^e^ | 1315.8±77.4^f^ | 3645.2±89.3^b^ | 3079.9±292.9^cd^ | 3557.5±110.7^bc^ | 3937.6±84.4^ab^ | 4356.7±101.3^a^ |
| **AGE60** | 1686.2±118.2^f^ | 1267.0±60.9^f^ | 2456.2±77.4^ef^ | 4113.0±223.3^e^ | 6647.2±446.6^d^ | 19005.9±913.5^b^ | 23674.5±751.4^a^ | 17163.7±643.3^b^ | 23616.0±194.7^a^ | 14649.1±1494.7^c^ |
| **RAW** | 984.4±150.1^e^ | 1452.2±194.7^bcd^ | 1306.0±60.9^d^ | 1374.3±29.3^cd^ | 1403.5±50.6^bcd^ | 1793.4±67.5^a^ | 1617.9±118.2^abc^ | 1686.2±73.6^ab^ | 1520.5±58.5^abcd^ | 1686.2±118.2^ab^ |

Values are expressed as mean ± SD (n=3). Means with different superscripts within rows are significantly different when analyzed by One-way ANOVA, and the Tukey test at *P ≤ 0.05*. Fermented garlic in Ethanol (FGE), fermented garlic in vinegar (FGV), fermented garlic in honey (FGH), aged garlic at 60°C/95% RH (AGED60), and fresh raw garlic (RAW) extracts.

***Sup* Table 8. Total phenolic content (TPC - mg GAE/g) of (A) FGE, (B) FGV, (C) FGH, (D) AGED60, and (E) RAW**

| **Total Phenolic Content of Processed Garlic Products - Total Phenolic Content (mg GAE/g)** | | | | | | | | | | |
| --- | --- | --- | --- | --- | --- | --- | --- | --- | --- | --- |
| **Garlic Sample** | **Processing Period** | | | | | | | | | |
|  | **Day 1** | **Week 1** | **Week 2** | **Week 3** | **Week 4** | **2 Months** | **3 Months** | **4 Months** | **5 Months** | **6 Months** |
| **FGE** | 667.5±12.2^bc^ | 399.9±8.6^d^ | 332.1±8.1^de^ | 335.2±4.1^de^ | 281.4±5.5^e^ | 744.5±111.7^ab^ | 576.2±4.9^c^ | 786.5±12.9^a^ | 587.5±6.7^c^ | 379.3±5.4^de^ |
| **FGV** | 656.8±21.6^c^ | 613.7±4.1^c^ | 643.9±23.1^c^ | 536.8±19.7^d^ | 766.5±10.9^b^ | 431.7±0.9^e^ | 395.2±8.6^ef^ | 524.5±5.6^d^ | 1033.7±40.5^a^ | 349.6±5.4^f^ |
| **FGH** | 667.5±24.7^c^ | 781.9±9.4^c^ | 825.0±14.6^c^ | 838.3±23.1^c^ | 645.0±24.5^c^ | 1438.3±267.2^b^ | 1512.2±87.7^b^ | 1571.7±46.6^ab^ | 1608.1±70.8^ab^ | 1815.8±6.2^a^ |
| **AGE60** | 642.4±4.7^e^ | 416.3±9.0^f^ | 611.6±9.4^ef^ | 918.8±10.2^d^ | 1366.5±13.8^c^ | 2771.1±87.1^b^ | 3679.3±24.7^a^ | 2815.2±172.4^b^ | 3771.6±89.4^a^ | 2798.8±23.3^b^ |
| **RAW** | 655.7±13.2^bc^ | 667.5±12.2^ab^ | 656.8±21.6^bc^ | 582.9±13.7^d^ | 609.6±4.4^cd^ | 642.4±4.7^bc^ | 675.2±29.9^ab^ | 667.5±24.7^ab^ | 708.5±12.4^a^ | 642.9±18.9^bc^ |

Values are expressed as mean ± SD (n=3). Means with different superscripts within rows are significantly different when analyzed by One-way ANOVA, and the Tukey test at *P ≤ 0.05*. Fermented garlic in Ethanol (FGE), fermented garlic in vinegar (FGV), fermented garlic in honey (FGH), aged garlic at 60°C/95% RH (AGED60), and fresh raw garlic (RAW) extracts.

**Sup Table 9. Fermented garlic in vinegar (FGV) antimicrobial activity**

| **Fermented Garlic in Vinegar (FGV)** | | | | | | | | | | |
| --- | --- | --- | --- | --- | --- | --- | --- | --- | --- | --- |
| **Test**  **Organisms** | **IZ (mm)** | | | | | | | | | |
|  | **C (+)** | **Week 1** | **Week 2** | **Week 3** | **Week 4** | **2 Months** | **3 Months** | **4 Months** | **5 Months** | **6 Months** |
| **A.** *E. coli* | 23.0±0.0^a^ | 21.3±1.2^a^ | 11.3±0.6^d^ | 15.3±1.2^bc^ | 11.0±0.0^d^ | 12.7±0.6^bcd^ | 12.0±0.0^cd^ | 16.0±3.0^b^ | 12.7±1.2^bcd^ | 14.0±0.0^bcd^ |
| MIC (mg/L) |  | 3.1 | 25 | 25 | 25 | 12.5 | 12.5 | 12.5 | 25 | 0 |
| **B.** *A. niger* | 12.3±0.6^c^ | 25.0±0.0^a^ | 17.3±1.2^b^ | 11.3±0.6^c^ | 12.0±0.0^e^ | 0.0±0.0^d^ | 0.0±0.0^d^ | 0.0±0.0^d^ | 0.0±0.0^d^ | 0.0±0.0^d^ |
| MIC (mg/L) |  | 8.8 | 187.5 | 375 | 250 | 500 | 375 | 500 | 500 | 250 |
| **C.** *P. guilliermondii* | 13.7±2.1^e^ | 25.7±0.6^b^ | 16.7±0.6^d^ | 13.0±1.0^e^ | 22.7±2.1^c^ | 10.3±0.6^f^ | 13.7±0.6^e^ | 13.7±0.6^e^ | 20.3±0.6^c^ | 30.0±0.0^a^ |
| MIC (mg/L) |  | 35.2 | 128.9 | 132.8 | 15.6 | 125.0 | 125.0 | 62.5 | 187.5 | 15.6 |

Values are expressed as mean ± SD (n=3). Means with different superscripts within rows are significantly different when analyzed by one-way ANOVA, and Tukey test at *P ≤ 0.05*. Fermented garlic in Ethanol (FGE), fermented garlic in vinegar (FGV), fermented garlic in honey (FGH), aged garlic at 60°C/95% RH (AGED60), and fresh raw garlic (RAW) extracts.

**Sup Table 10. Fermented garlic in honey (FGH) antimicrobial activity.**

| **Fermented Garlic in Honey (FGH)** | | | | | | | | | | |
| --- | --- | --- | --- | --- | --- | --- | --- | --- | --- | --- |
| **Test**  **Organisms** | **IZ (mm)** | | | | | | | | | |
|  | **C (+)** | **Week 1** | **Week 2** | **Week 3** | **Week 4** | **2 Months** | **3 Months** | **4 Months** | **5 Months** | **6 Months** |
| **A.** *E. coli* | 23.0±0.0^b^ | 26.7±1.5^a^ | 24.7±1.2^ab^ | 19.0±1.0^c^ | 11.7±0.6^d^ | 10.7±0.6^d^ | 11.0±0.0^d^ | 11.3±1.2^d^ | 11.0±0.0^d^ | 11.3±0.0^d^ |
| MIC (mg/L) |  | 4 | 4 | 250 | 250 | 250 | 250 | 63 | 250 | 250 |
| **B.** *A. niger* | 12.3±0.6^c^ | 25.0±0.0^a^ | 25.0±0.0^a^ | 15.7±2.5^b^ | 11.0±0.0^c^ | 11.7±0.6^c^ | 23.3±2.1^a^ | 16.7±0.6^b^ | 23.0±1.0^a^ | 18.0±0.0^b^ |
| MIC (mg/L) |  | 5 | 9 | 500 | 188 | 375 | 500 | 500 | 500 | 500 |
| **C.** *P. guilliermondii* | 12.0±1.0^de^ | 18.7±0.6^ab^ | 11.7±0.6^e^ | 12.7±1.2^e^ | 13.7±2.1^cde^ | 21.7±0.6^a^ | 16.7±3.1^bcd^ | 17.3±1.2^bc^ | 14.0±0.0^cde^ | 12.0±0.0^e^ |
| MIC (mg/L) |  | 133 | 64 | 39 | 70 | 16 | 23 | 63 | 188 | 31 |

Values are expressed as mean ± SD (n=3). Means with different superscripts within rows are significantly different when analyzed by One-way ANOVA, and the Tukey test at *P ≤ 0.05*. Fermented garlic in Ethanol (FGE), fermented garlic in vinegar (FGV), fermented garlic in honey (FGH), aged garlic at 60°C/95% RH (AGED60, and fresh raw garlic (RAW) extracts.

**Sup Table 11. Aged black garlic at 60°C (AGED60) antimicrobial activity.**

| **Aged Black Garlic at 60°C (AGE60°C)** | | | | | | | | | | |
| --- | --- | --- | --- | --- | --- | --- | --- | --- | --- | --- |
| **Test**  **Organisms** | **IZ (mm)** | | | | | | | | | |
|  | **C (+)** | **Week 1** | **Week 2** | **Week 3** | **Week 4** | **2 Months** | **3 Months** | **4 Months** | **5 Months** | **6 Months** |
| **A.** *E. coli* | 23.0±0.0^a^ | 13.7±0.6^bc^ | 14.3±0.6^b^ | 15.7±0.6^b^ | 11.7±0.6^c^ | 14.0±1.7^bc^ | 13.7±0.6^bc^ | 16.0±1.0^b^ | 13.7±1.2^bc^ | 14.7±0.6^b^ |
| MIC (mg/L) |  | 0 | 125 | 63 | 31 | 25 | 6 | 13 | 13 | 0 |
| **B.** *A. niger* | 13.0±1.7^cd^ | 17.7±0.6^a^ | 16.0±0.0^b^ | 12.3±0.6^c^ | 11.3±0.6^d^ | 0.0±0.0^e^ | 0.0±0.0^e^ | 0.0±0.0^e^ | 0.0±0.0^e^ | 0.0±0.0^e^ |
| MIC (mg/L) |  | 281 | 500 | 500 | 313 | 100 | 100 | 100 | 100 | 500 |
| **C.** *P. guilliermondii* | 12.0±1.0^d^ | 12.3±0.6^d^ | 24.3±1.2^a^ | 12.3±0.6^d^ | 13.3±1.2^d^ | 17.7±0.6^bc^ | 19.7±1.2^b^ | 13.3±0.6^d^ | 12.7±0.6^d^ | 16.0±0.0^c^ |
| MIC (mg/L) |  | 133 | 156 | 188 | 47 | 125 | 63 | 38 | 9 | 31 |

Values are expressed as mean ± SD (n=3). Means with different superscripts within rows are significantly different when analyzed by One-way ANOVA, and the Tukey test at *P ≤ 0.05*. Fermented garlic in Ethanol (FGE), fermented garlic in vinegar (FGV), fermented garlic in honey (FGH), aged garlic at 60°C/95% RH (AGED60), and fresh raw garlic (RAW) extracts.

**Sup Table 12. Fresh raw garlic (RAW) antimicrobial activity.**

| **Fresh Raw Garlic (RAW)** | | | | | | | | | | |
| --- | --- | --- | --- | --- | --- | --- | --- | --- | --- | --- |
| **Test**  **Organisms** | **IZ (mm)** | | | | | | | | | |
|  | **C (+)** | **Week 1** | **Week 2** | **Week 3** | **Week 4** | **2 Months** | **3 Months** | **4 Months** | **5 Months** | **6 Months** |
| **A.** *E. coli* | 23.0±0.0^b^ | 26.7±1.5^a^ | 25.7±1.2^a^ | 18.7±1.2^cd^ | 14.0±0.0^g^ | 16.3±0.6^ef^ | 16.0±0.0^fg^ | 18.3±0.6^de^ | 20.0±0.0^cd^ | 20.7±0.6^c^ |
| MIC (mg/L) |  | 6 | 6 | 6 | 6 | 6 | 13 | 13 | 13 | 13 |
| **B.** *A. niger* | 12.7±1.2^b^ | 25.0±0.0^a^ | 23.7±2.3^a^ | 22.3±1.5^a^ | 23.3±2.1^a^ | 14.7±2.1^b^ | 0.0±0.0^c^ | 0.0±0.0^c^ | 0.0±0.0^c^ | 0.0±0.0^c^ |
| MIC (mg/L) |  | 32 | 32 | 32 | 32 | 32 | 14 | 14 | 14 | 14 |
| **C.** *P. guilliermondii* | 12.3±1.5^c^ | 14.0±2.6^c^ | 20.7±0.6^b^ | 12.3±1.5^c^ | 14.0±2.0^c^ | 15.0±0.0^c^ | 12.7±1.2^c^ | 27.3±0.6^a^ | 24.0±1.0^ab^ | 14.3±0.6^c^ |
| MIC (mg/L) |  | 66 | 64 | 64 | 64 | 125 | 125 | 156 | 250 | 250 |

Values are expressed as mean ± SD (n=3). Means with different superscripts within rows are significantly different when analyzed by One-way ANOVA, and the Tukey test at *P ≤ 0.05*. Fermented garlic in Ethanol (FGE), fermented garlic in vinegar (FGV), fermented garlic in honey (FGH), aged garlic at 60°C/95% RH (AGED60), and fresh raw garlic (RAW) extracts.
